# Recent Selective Sweeps in North American *Drosophila melanogaster* Show Signatures of Soft Sweeps

**DOI:** 10.1371/journal.pgen.1005004

**Published:** 2015-02-23

**Authors:** Nandita R. Garud, Philipp W. Messer, Erkan O. Buzbas, Dmitri A. Petrov

**Affiliations:** 1 Department of Genetics, Stanford University, Stanford, California, United States of America; 2 Department of Biology, Stanford University, Stanford, California, United States of America; 3 Department of Biological Statistics and Computational Biology, Cornell University, Ithaca, New York, United States of America; 4 Department of Statistical Science, University of Idaho, Moscow, Idaho, United States of America; The University of North Carolina at Chapel Hill, UNITED STATES

## Abstract

Adaptation from standing genetic variation or recurrent *de novo* mutation in large populations should commonly generate soft rather than hard selective sweeps. In contrast to a hard selective sweep, in which a single adaptive haplotype rises to high population frequency, in a soft selective sweep multiple adaptive haplotypes sweep through the population simultaneously, producing distinct patterns of genetic variation in the vicinity of the adaptive site. Current statistical methods were expressly designed to detect hard sweeps and most lack power to detect soft sweeps. This is particularly unfortunate for the study of adaptation in species such as *Drosophila melanogaster*, where all three confirmed cases of recent adaptation resulted in soft selective sweeps and where there is evidence that the effective population size relevant for recent and strong adaptation is large enough to generate soft sweeps even when adaptation requires mutation at a specific single site at a locus. Here, we develop a statistical test based on a measure of haplotype homozygosity (H12) that is capable of detecting both hard and soft sweeps with similar power. We use H12 to identify multiple genomic regions that have undergone recent and strong adaptation in a large population sample of fully sequenced *Drosophila melanogaster* strains from the Drosophila Genetic Reference Panel (DGRP). Visual inspection of the top 50 candidates reveals that in all cases multiple haplotypes are present at high frequencies, consistent with signatures of soft sweeps. We further develop a second haplotype homozygosity statistic (H2/H1) that, in combination with H12, is capable of differentiating hard from soft sweeps. Surprisingly, we find that the H12 and H2/H1 values for all top 50 peaks are much more easily generated by soft rather than hard sweeps. We discuss the implications of these results for the study of adaptation in Drosophila and in species with large census population sizes.

## Author Summary

Evolutionary adaptation is a process in which beneficial mutations increase in frequency in response to selective pressures. If these mutations were previously rare or absent from the population, adaptation should generate a characteristic signature in the genetic diversity around the adaptive locus, known as a selective sweep. Such selective sweeps can be distinguished into *hard* selective sweeps, where only a single adaptive mutation rises in frequency, or *soft* selective sweeps, where multiple adaptive mutations at the same locus sweep through the population simultaneously. Here we design a new statistical method that can identify both hard and soft sweeps in population genomic data and apply this method to a *Drosophila melanogaster* population genomic dataset consisting of 145 sequenced strains collected in North Carolina. We find that selective sweeps were abundant in the recent history of this population. Interestingly, we also find that practically all of the strongest and most recent sweeps show patterns that are more consistent with soft rather than hard sweeps. We discuss the implications of these findings for the discovery and quantification of adaptation from population genomic data in Drosophila and other species with large population sizes.

## Introduction

The ability to identify genomic loci subject to recent positive selection is essential for our efforts to uncover the genetic basis of phenotypic evolution and to understand the overall role of adaptation in molecular evolution. The fruit fly *Drosophila melanogaster* is one of the classic model organisms for studying the molecular bases and signatures of adaptation. Recent studies have provided evidence for pervasive molecular adaptation in this species, suggesting that approximately 50% of the amino acid changing substitutions, and similarly large proportions of non-coding substitutions, were adaptive [1,2,3,4,5,6,7,8,9]. There is also evidence that at least some of these adaptive events were driven by strong positive selection (~1% or larger), depleting levels of genetic variation on scales of tens of thousands of base pairs in length [10,11].

If adaptation in *D*. *melanogaster* is indeed common and often driven by strong selection, it should be possible to detect genomic signatures of recent and strong adaptation [12,13,14]. Three cases of recent and strong adaptation in *D*. *melanogaster* are well documented and can inform our intuitions about the expected genomic signatures of such adaptive events. First, resistance to the most commonly used pesticides, carbamates and organophosphates, is known to be largely due to three point mutations at highly conserved sites in the gene *Ace*, which encodes the neuronal enzyme Acetylcholinesterase [15,16,17]. Second, resistance to DDT evolved *via* a series of adaptive events that included insertion of an *Accord* transposon in the 5’ regulatory region of the gene *Cyp6g1*, duplication of the locus, and additional transposable element insertions into the locus [18,19]. Finally, increased resistance to infection by the sigma virus, as well as resistance to certain organophosphates, has been associated with a transposable element insertion in the protein-coding region of the gene *CHKov1* [20,21].

In-depth population genetic studies [17,19,21] of adaptation at these loci revealed that in all three cases adaptation failed to produce classic hard selective sweeps, but instead generated patterns compatible with soft sweeps. In a hard selective sweep, a single adaptive haplotype rises in frequency and removes genetic diversity in the vicinity of the adaptive locus [22,23,24]. In contrast, in a soft sweep multiple adaptive alleles present in the population as standing genetic variation (SGV) or entering as multiple *de novo* adaptive mutations increase in frequency virtually simultaneously bringing multiple haplotypes to high frequency [25,26,27,28,29]. In the cases of *Ace* and *Cyp6g1*, soft sweeps involved multiple *de novo* mutations [17,19,21] that arose after the introduction of pesticides, whereas in the case of *CHKov1*, a soft sweep arose in out-of-African populations from standing genetic variation (SGV) [17,19,21] present at low frequencies in the ancestral African population [20,21].

Unfortunately, most scans for selective sweeps in population genomic data have been designed to detect hard selective sweeps (although see [30]) and focus on such signatures as a dip in neutral diversity around the selected site [22,24,31], an excess of low or high-frequency alleles in the frequency spectrum of polymorphisms surrounding the selected site (i.e. Tajima’s *D*, Fay and Wu’s *H*, and Sweepfinder) [32,33,34,35,36], the presence of a single common haplotype [37], or the observation of a long and unusually frequent haplotype (*iHS*) [36,38,39,40]. In a soft sweep, however, multiple haplotypes linked to the selected locus can rise to high frequency and levels of diversity and allele frequency spectra should therefore be perturbed to a lesser extent than in a hard sweep. As a result, methods based on the levels and frequency distributions of neutral diversity have low power to detect soft sweeps [13,28,41,42].

Some genomic signatures do have power to detect both hard and soft sweeps. In particular, linkage disequilibrium (LD) measured between pairs of sites or as haplotype homozygosity should be elevated in both hard and soft sweeps. This expectation holds for hard sweeps and for soft sweeps that are not too soft, that is soft sweeps that have such a large number of independent haplotypes bearing adaptive alleles that linkage disequilibrium is no longer elevated beyond neutral expectations [41,43].

Given that none of the described cases of adaptation at *Ace*, *Cyp6g1*, and *CHKov1* produced hard sweeps, it is possible that additional cases of recent selective sweeps in *D*. *melanogaster* remain to be discovered. Here we develop a statistical test based on modified haplotype homozygosity for detecting both hard and soft selective sweeps in population genomic data. We apply this test in a genome-wide scan in a North American population of *D*. *melanogaster* using the Drosophila Genetic Reference Panel (DGRP) data set [44], consisting of 162 fully sequenced isogenic strains from a North Carolina population. Our scan recovers the three known soft sweeps at *Ace*, *Cyp6g1*, and *CHKov1*, and identifies a large number of additional recent and strong selective sweeps. We develop an additional haplotype homozygosity statistic that can distinguish hard from soft sweeps and argue that the haplotype frequency spectra at the top 50 candidate sweeps are best explained by soft selective sweeps.

## Results

### Slow decay of linkage disequilibrium in the DGRP data

In this paper, we develop a set of new statistics for the detection and characterization of positive selection based on measurements of haplotype homozygosity in a predefined window. Our reasoning in developing these statistics is that haplotype homozygosity, defined as a sum of squares of the frequencies of identical haplotypes in a window, should be a sensitive statistic for the detection of both hard and soft sweeps, as long as the window is large enough that neutral demographic processes are unlikely to elevate haplotype homozygosity by chance [41,43]. At the same time, the window must not be so large that even strong sweeps can no longer generate frequent haplotypes spanning the whole window.

**Fig 1:**
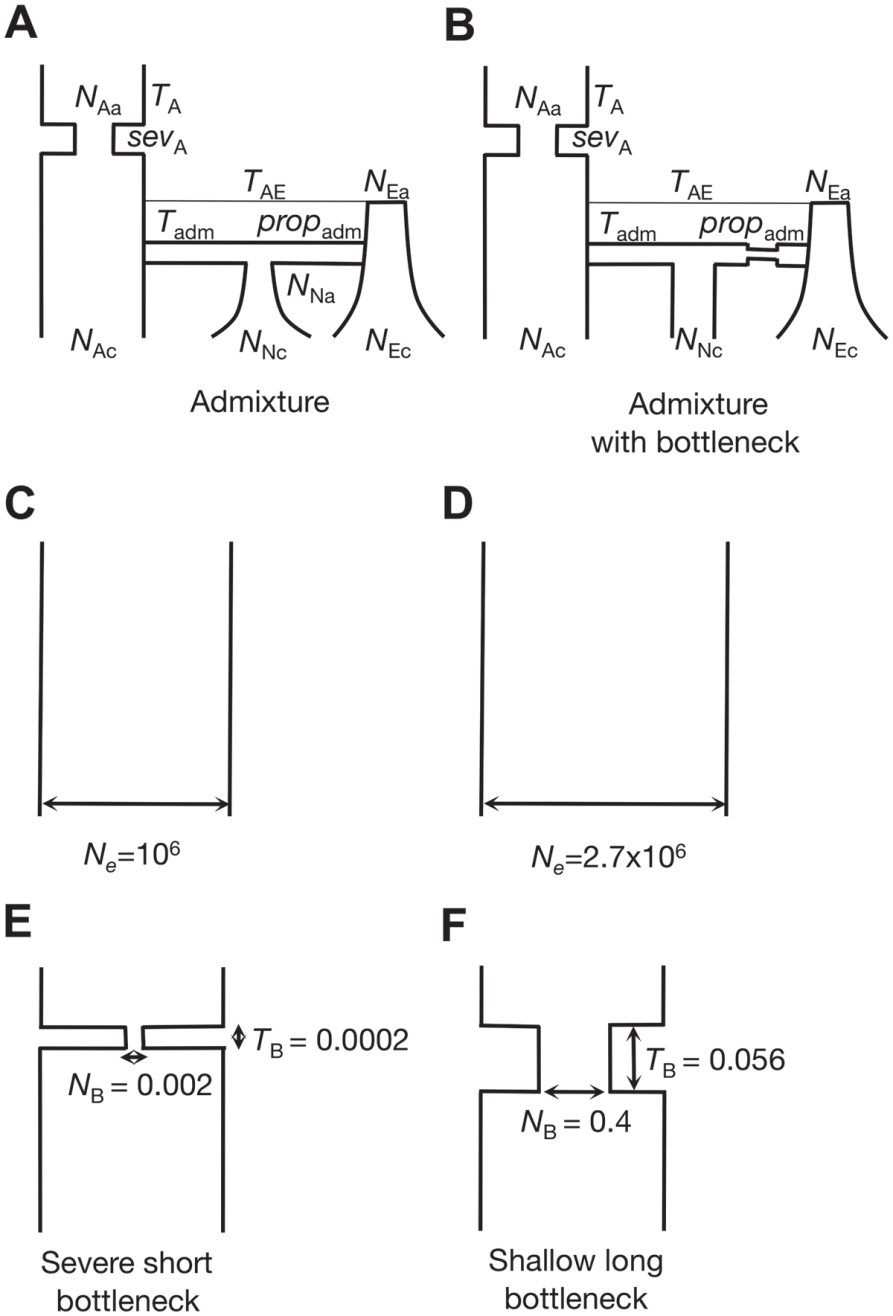
Neutral demographic models. We considered six neutral demographic models for the North American *D*. *melanogaster* population: (A) An admixture model as proposed by Duchen *et al*. [45]. (B) An admixture model with the European population undergoing a bottleneck. This model was also tested by Duchen *et al*. [45], but the authors found it to have a poor fit. See S1 Table for parameter estimates and symbol explanations for models A and B. (C) A constant *N*_*e*_ = 10^6^ model. (D) A constant *N*_*e*_ = 2.7x10^6^ model fit to Watterson’s *θ*_W_ measured in short intron autosomal polymorphism data from the DGRP data set. (E) A severe short bottleneck model and (F) a shallow long bottleneck model fit to short intron regions in the DGRP data set using DaDi [47]. See S2 Table for parameter estimates for models E and F. All models except for the constant *N*_*e*_ = 10^6^ model fit the DGRP short intron data in terms of *S* and *π* (S3 Table).

In order to determine an appropriate window length for the measurement of haplotype homozygosity in the DGRP data set, we first assessed the length scale of linkage disequilibrium decay expected in the DGRP data under a range of neutral demographic models for North American *D*. *melanogaster*. This length scale should roughly correspond to the window size over which we are unlikely to observe substantial haplotype structure by chance. We considered six demographic models (Fig. 1). The first demographic model is an admixture model of the North American *D*. *melanogaster* population proposed by Duchen *et al*. [45]. In this model, the North American population was co-founded by flies from Africa and Europe 3.05×10^–4^ *N*_*e*_ generations ago (where *N*_*e*_ ≈ 5x10^6^). The second model is a modified admixture model, also proposed by Duchen *et al*. [45], in which the founding European population underwent a bottleneck before the admixture event (see S1 Table for complete parameterizations of both admixture models). The third model has a constant effective population size of *N*_*e*_ = 10^6^ [46], which we considered for its simplicity, computational feasibility and, as we will argue below, its conservativeness for the purposes of detecting selective sweeps using our approach in the DGRP data. The fourth model is a constant *N*_*e*_ = 2.7x10^6^ demographic model fit to Watterson’s *θ*_W_ estimated from short intron autosomal polymorphism data from the DGRP dataset (Methods). Finally, we fit a family of out-of-Africa bottleneck models to short intron regions in the DGRP data set using DaDi [47] (S2 Table) (Methods). The two bottleneck models we ultimately used are a severe but short bottleneck model (*N*_*B*_ = 0.002, *T*_*B*_ = 0.0002) and a shallow but long bottleneck model (*N*_*B*_ = 0.4, *T*_*B*_ = 0.0560), both of which fit the data equally well among a range of other inferred bottleneck models (see S1 Fig. for parameterization). All models except for the constant *N*_*e*_ = 10^6^ model fit the DGRP short intron data in terms of the number of segregating sites (*S*) and pairwise nucleotide diversity (*π*) (S3 Table).

**Fig 2:**
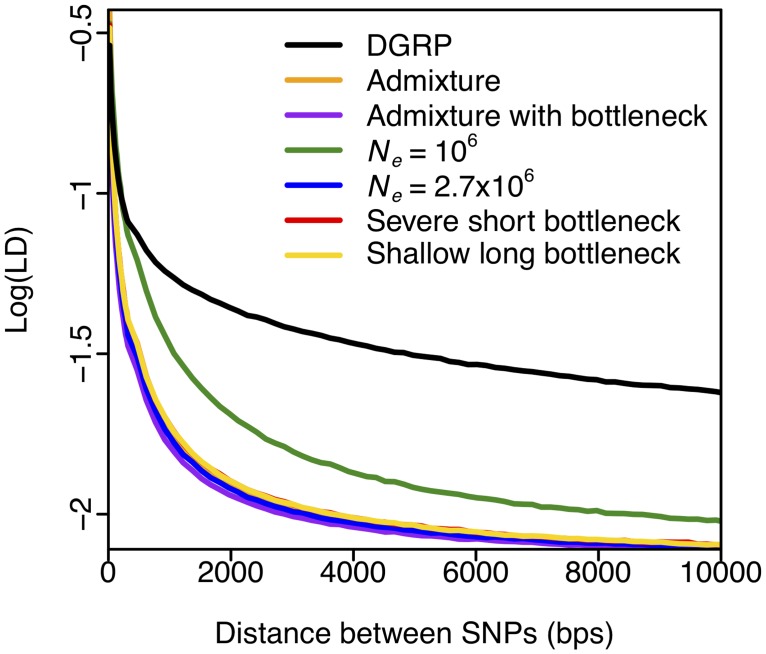
Elevated long-range LD in DGRP. LD in DGRP data is elevated as compared to any neutral demographic model, especially for long distances. Pairwise LD was calculated in DGRP data for regions of the *D*. *melanogaster* genome with *ρ* ≥ 5×10^–7^ cM/bp. Neutral demographic simulations were generated with *ρ* = 5×10^–7^ cM/bp. Pairwise LD was averaged over 3×10^4^ simulations in each neutral demographic scenario.

We compared the decay in pair-wise LD in the DGRP data at distances from a few base pairs to 10 kb with the expectations under each of the six demographic models using parameters relevant for our subsequent analysis of the DGRP data (Fig. 2). Specifically, we matched the sample depth of the DGRP data set (145 strains after quality control) and assumed a mutation rate (μ) of 10^–9^ events/bp per generation [48] and a recombination rate (*ρ*) of 5×10^–7^ centimorgans/bp (cM/bp) [49]. In the DGRP data analysis below, we exclude regions with a low recombination rate (*ρ* < 5x10^–7^ cM/bp). The use of *ρ* = 5x10^–7^ cM/bp should therefore generate higher LD in simulations than in the DGRP data and thus should be conservative for the purposes of defining the expected length scale of LD decay.

Fig. 2 shows that LD in the DGRP data is elevated beyond neutral expectations at all length scales (consistent with the observations in [50]), and dramatically so at the 10 kb length scale. The elevation in LD observed in the data is indicative of either linked positive selection driving haplotypes to high frequency, a lack of fit of current demographic models to the data, or both. Simulations under the most realistic demographic model, admixture [45], have the fastest decay in LD (S2 Fig.). This is likely because admixture models with two bottlenecks that are fit to diversity statistics generate more haplotypes compared to single bottleneck models, since the same haplotype is unlikely to be sampled independently in both bottlenecked ancestral populations. In contrast, LD under the constant *N*_*e*_ = 10^6^ demographic scenario decays slower than in any other demographic scenario, as expected given that this model has the smallest effective population size.

Fig. 2 suggests that windows of 10 kb are large enough that neutral demography is unlikely to generate high values of LD and elevate haplotype homozygosity by chance, and should thus prevent a high rate of false positives. At the same time, the use of 10 kb windows for the measurement of haplotype homozygosity should still allow us to detect many reasonably strong sweeps, including the known cases of recent adaptation. The footprint of a hard selective sweep extends over approximately *s*/[log(*N*_*e*_ *s*)*ρ*] basepairs, where *s* is the selection strength, *N*_*e*_ the population size, and *ρ* the recombination rate [22,23,51]. Sweeps with a selection coefficient of *s* = 0.05% or greater are thus likely to generate sweeps that span 10 kb windows in areas with recombination rate of 5×10^–7^ cM/bp. As the recombination rate increases, only selective sweeps with *s* > 0.05% should be observed in the 10 kb windows. Genomic analyses have suggested that adaptation in Drosophila is likely associated with a range of selection strengths, including values of ~1% [7,8,10] or greater as observed at *Ace*, *Cyp6g1*, and *CHKov1*. Our use of 10 kb windows in the rest of the analysis should thus bias the analysis toward detecting the cases of strongest adaptation in Drosophila.

### Haplotype spectra expectations under selective sweeps of varying softness

We investigated haplotype spectra in simulations of neutral demography and both hard and soft selective sweeps arising from *de novo* mutations as well as SGV. For all haplotype spectra and homozygosity analyses in this paper we use windows of 400 SNPs, corresponding roughly to 10 kb in the DGRP data (Fig. 2). Haplotypes within a 400 SNP window are grouped together if they are identical at all SNPs in the window. We fixed the number of SNPs in a window to eliminate variability in the haplotype spectra due to varying numbers of SNPs.

The lower SNP density of the constant *N*_*e*_ = 10^6^ model (S3 Table) effectively increases the size of the analysis window in terms of the number of base pairs when defining the windows in terms of the number of SNPs. Thus, the constant *N*_*e*_ = 10^6^ model should reduce the rate of false positives because the recombination rate under this model is artificially increased. We therefore use the constant *N*_*e*_ = 10^6^ model for the subsequent simulations of neutrality and selective sweeps.

To visualize sample haplotype frequency spectra, we simulated incomplete and complete sweeps with frequencies of the adaptive mutation (*PF*) at 0.5 or 1 at the time when selection ceased. (Note that below we will investigate a large number of scenarios, focusing on the effects of varying selection strength and the decay of sweep signatures with time). The number of independent haplotypes that rise in frequency simultaneously in soft sweeps—we call this “softness” of a sweep—should increase either (i) when the rate of mutation to *de novo* adaptive alleles at a locus becomes higher and multiple alleles arise and establish after the onset of selection at a higher rate, or (ii) when adaptation uses SGV with previously neutral or deleterious alleles that are present at higher frequency at the onset of selection [27,29]. More specifically, for sweeps arising from multiple *de novo* mutations, Pennings and Hermisson [29] showed that the key population genetic parameter that determines the softness of the sweep is *θ*_A_ = 4*N*_*e*_μ_A_, proportional to the product of *N*_*e*_, the variance effective population size estimated over the period relevant for adaptation [14,52], and μ_A_, the mutation rate toward adaptive alleles at a locus per individual per generation [14]. The mutation-limited regime with hard sweeps corresponds to *θ*_A_ << 1, whereas *θ*_A_ > 1 specifies the non-mutation-limited regime with primarily soft sweeps. As *θ*_A_ becomes larger, the sweeps become softer as more haplotypes increase in frequency simultaneously [29]. In the case of sweeps arising from SGV, the softness of a sweep is governed by the starting partial frequency of the adaptive allele in the population prior to the onset of selection. For any given rate of recombination, adaptive alleles starting at a higher frequency at the onset of selection should be older and should thus be present on more distinct haplotypes and give rise to softer sweeps [27].

**Fig 3:**
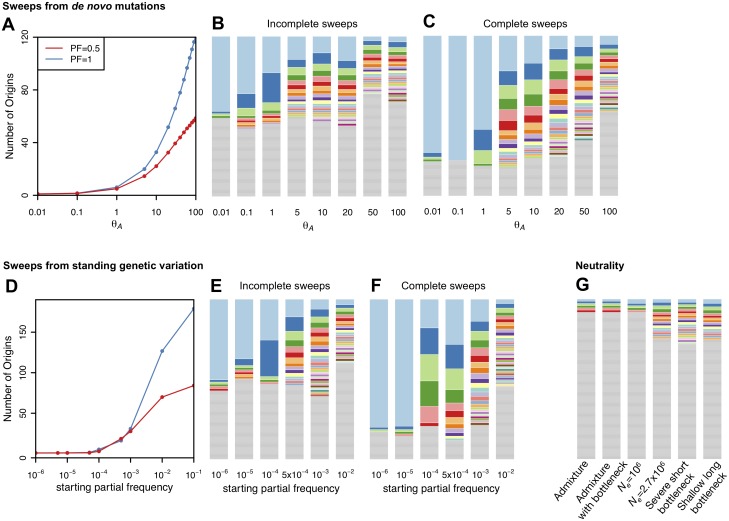
Number of adaptive haplotypes in sweeps of varying softness. The number of origins of adaptive mutations on unique haplotype backgrounds was measured in simulated sweeps of varying softness arising from (A) *de novo* mutations with *θ*_A_ values ranging from 10^–2^ to 10^2^ and (D) SGV with starting frequencies ranging from 10^–6^ to 10^–1^. Sweeps were simulated under a constant *N*_*e*_ = 10^6^ demographic model with a recombination rate of 5×10^–7^ cM/bp, selection strength of *s* = 0.01, partial frequency of the adaptive allele after selection has ceased of *PF* = 1 and 0.5, and in sample sizes of 145 individuals. 1000 simulations were averaged for each data point. Additionally we show sample haplotype frequency spectra for (B) incomplete and (C) complete sweeps arising from *de novo* mutations as well as (E) incomplete and (F) complete sweeps arising from SGV. In (G) we show haplotype frequency spectra for a random simulation under the six neutral models considered in this paper. The height of the first bar (light blue) in each frequency spectrum indicates the frequency of the most prevalent haplotype in the sample of 145 individuals, and heights of subsequent colored bars indicate the frequency of the second, third, and so on most frequent haplotypes in a sample. Grey bars indicate singletons. Sweeps generated with a low *θ*_A_ or low starting partial frequency of the adaptive allele prior to the onset of selection have one frequent haplotype in the sample and look hard. In contrast, sweeps look increasingly soft as the *θ*_A_ or starting partial frequency of the adaptive allele prior to the onset of selection increase and there are multiple frequent haplotypes in the sample.

As can be seen in Fig. 3, most haplotypes in neutral demographic scenarios are unique in our 400 SNP windows, whereas selective sweeps can generate multiple haplotypes at substantial frequencies. Our plot of the haplotype frequency spectra and the expected numbers of adaptive haplotypes show that sweeps arising from *de novo* mutations become soft with multiple frequent haplotypes in the sample when *θ*_A_ ≥ 1. Sweeps from SGV become soft when the starting partial frequency of the adaptive allele prior to the onset of selection is ≥ 10^–4^ (100 alleles in the population). In both cases, sweeps become monotonically softer as *θ*_A_ increases or, respectively, the starting partial frequency of the adaptive allele becomes higher. These results conform to the expectations derived in [29].

### Definitions of haplotype homozygosity statistics H1, H12, and H123

**Fig 4:**
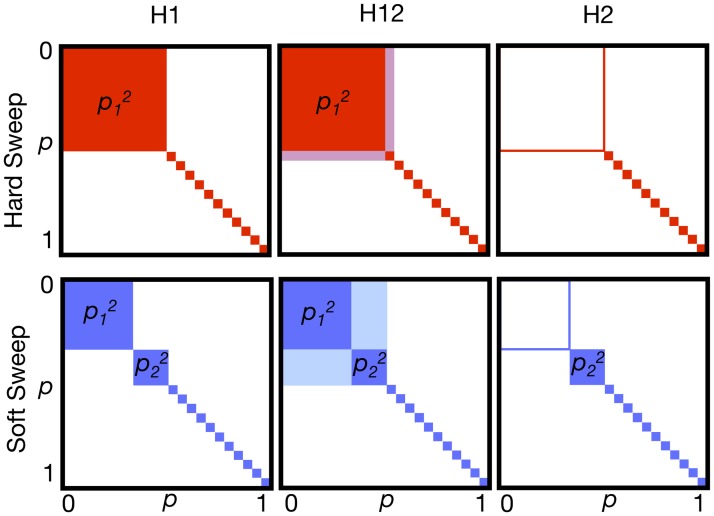
Haplotype homozygosity statistics. Depicted are squares of haplotype frequencies for hard (red) and soft (blue) sweeps. Each edge of the square represents haplotype frequencies ranging from 0 to 1. The top row shows incomplete hard sweeps with one prevalent haplotype present in the population at frequency *p*_*1*_, and all other haplotypes present as singletons. The bottom row shows incomplete soft sweeps with one primary haplotype with frequency *p*_*1*_ and a second, less abundant haplotype at frequency *p*_*2*_, with the remaining haplotypes present as singletons. H1 is the sum of the squares of frequencies of each haplotype in a sample and corresponds to the total colored area. Hard sweeps are expected to have a higher H1 value than soft sweeps. In H12, the first and second most abundant haplotype frequencies in a sample are combined into a single combined haplotype frequency and then homozygosity is recalculated using this revised haplotype frequency distribution. By combining the first and second most abundant haplotypes into a single group, H12 should have more similar power to detect hard and soft sweeps than H1. H2 is the haplotype homozygosity calculated after excluding the most abundant haplotype. H2 is expected to be larger for soft sweeps than for hard sweeps. We ultimately use the ratio H2/H1 to differentiate between hard and soft sweeps as we expect this ratio to have even greater discriminatory power than H2 alone.

The increase of haplotype population frequencies in both hard and soft sweeps can be captured using haplotype homozygosity [30,39,41]. If *p*_*i*_ is the frequency of the *i* ^th^ most common haplotype in a sample, and *n* is the number of observed haplotypes, then haplotype homozygosity is defined as H1 = Σ_*i* = 1, …n_ *p*_*i*_^2^. We can expect H1 to be particularly high for hard sweeps, with only one adaptive haplotype at high frequency in the sample (Fig. 4A). Thus, H1 is an intuitive candidate for a test of neutrality versus hard sweeps, where the test rejects neutrality for high values of H1. A test based on H1 may also have acceptable power to detect soft sweeps in which only a few haplotypes in the population are present at high frequency. However, as sweeps become softer and the number of sweeping haplotypes increases, the relative contribution of individual haplotypes towards the overall H1 value decreases, and the power of a test based on H1 is expected to decrease.

To have a better ability to detect hard and soft sweeps using homozygosity statistics, we developed a modified homozygosity statistic, H12 = (*p*_1_ + *p*_2_)^2^ + Σ_*i*>2_ *p*_*i*_^2^ = H1 + 2*p*_1_ *p*_2_, in which the frequencies of the first and the second most common haplotype are combined into a single frequency (Fig. 4B). A statistical test based on H12 is expected to be more powerful in detecting soft sweeps than H1 because it combines frequencies of two similarly abundant haplotypes into a single frequency, whereas for hard sweeps the combination of the frequencies of the first and second most abundant haplotypes should not change haplotype homozygosity substantially [53]. We also considered a third test statistic, H123, which combines frequencies of the three most prevalent haplotypes in a sample into a single haplotype and then computes homozygosity. We will primarily employ H12 in subsequent analyses but will consider the effects of using H1 and H123 briefly as well.

### Ability of H12 to detect selective sweeps of varying softness

To assess the ability of H12 to detect sweeps of varying softness and to distinguish positive selection from neutrality, we measured H12 in simulated sweeps arising from both *de novo* mutations and SGV while varying *s*, *PF*, and the time since the end of the sweep, *T*_*E*_, measured in units of 4*N*_*e*_ generations in order to model the decay of a sweep through recombination and mutation events over time. We first investigate the behavior of H12 under different selective regimes and then investigate its power in comparison with the popular haplotype statistic *iHS*.

**Fig 5:**
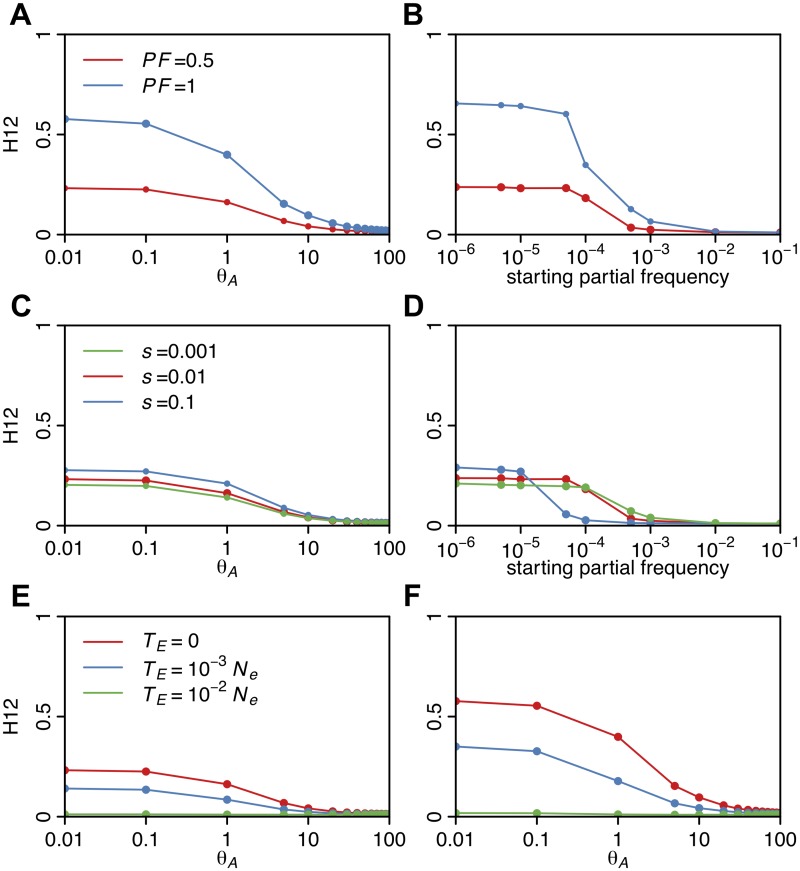
H12 values in sweeps of varying softness. H12 values were measured in simulated sweeps arising from (A) *de novo* mutations with *θ*_A_ values ranging from 10^–2^ to 10^2^ and (B) SGV with starting frequencies ranging from 10^–6^ to 10^–1^. Sweeps were simulated under a constant *N*_*e*_ = 10^6^ demographic model with a recombination rate of 5×10^–7^ cM/bp, selection strength of *s* = 0.01, ending partial frequencies of the adaptive allele after selection has ceased, *PF* = 1 and 0.5, and in samples of 145 individuals. Each data point was averaged over 1000 simulations. H12 values rapidly decline as the softness of a sweep increases and as the ending partial frequency of the adaptive allele decreases. In (C) and (D), *s* was varied while keeping *PF* constant at 0.5 for sweeps from *de novo* mutations and SGV, respectively. H12 values increase as *s* increases, though for very weak *s* we observe a ‘hardening’ of sweeps where fewer adaptive alleles reach establishment frequency. In (E) and (F), the time since selection ended (*T*_*E*_) was varied for incomplete (*PF* = 0.5) and complete (*PF* = 1) sweeps respectively while keeping *s* constant at 0.01. As the age of a sweep increases, sweep signatures decay and H12 loses power.

Fig. 5A shows that for complete and incomplete sweeps with *s* = 0.01 and *T*_*E*_ = 0, H12 monotonically decreases as a function of *θ*_A_ over the interval from 10^–2^ to 10^2^. When *θ*_A_ ≤ 0.5, many sweeps are hard and H12 values are high. When *θ*_A_ ≈ 1, and practically all sweeps are soft, but not yet extremely soft, H12 retains much of its power. However, for *θ*_A_ > 10, where sweeps are extremely soft, H12 decreases substantially. Similarly, H12 is maximized when the starting frequency of the allele is 10^–6^ (one copy of the allele in the population generating hard sweeps from SGV) and becomes very small as the frequency of the adaptive allele increases beyond >10^-3^ (>1000 copies of the allele in the population) (Fig. 5B). Therefore, H12 has reasonable power to detect soft sweeps in samples of hundreds of haplotypes, as long as they are not extremely soft, but remains somewhat biased in favor of detecting hard sweeps.

H12 also increases as the ending partial frequency of the adaptive allele after selection ceased (*PF*) increases from 0.5 to 1 (Fig. 5A and 5B) and as the selection strength increases from 0.001 to 0.1 (Fig. 5C and 5D). We observe that sweeps arising from SGV with low selection coefficients have lower H12 values (Fig. 5D). This is most likely because such weak sweeps are effectively harder: as more of the haplotypes fail to establish, fewer haplotypes end up sweeping in the population leading to higher values of haplotype homozygosity. Fig. 5E and 5F further show that incomplete and complete sweeps decay with time due to recombination and mutation events, resulting in monotonically decreasing values of H12 with time. Overall this analysis demonstrates that H12 has most power to detect recent sweeps driven by strong selection.

We also assessed the ability of H12 to detect selective sweeps as compared to H1 and H123 by calculating the values of H1, H12, and H123 for sweeps generated under the parameters *s* = 0.01, *T*_*E*_ = 0 and *PF* = 0.5. H12 consistently, albeit modestly, increases the homozygosity for younger soft sweeps as compared to H1 (S3 Fig.). The increase in homozygosity using H123 is marginal relative to homozygosity levels achieved by H12, so we chose not to use this statistic in our study.

Finally, we compared the abilities of H12 and *iHS* (integrated haplotype score), a haplotype-based statistic designed to detect incomplete hard sweeps [39,40], to detect both hard and soft sweeps. We created receiving operator characteristic (ROC) curves [54], which plot the true positive rate (TPR) of correctly rejecting neutrality in favor of a sweep (hard or soft) given that a sweep has occurred versus the false positive rate (FPR) of inferring a selective sweep, when in fact a sweep has not occurred.

In our simulations of selective sweeps we used *θ*_A_ = 0.01 as a proxy for scenarios generating almost exclusively hard sweeps, and *θ*_A_ = 10 as a proxy for scenarios generating almost exclusively soft sweeps. We chose *θ*_A_ = 10 for soft sweeps because this is the highest *θ*_A_ value with which H12 can still detect sweeps before substantially losing power given our window size of 400 SNPs and sample size of 145. Note that for soft sweeps with a lower value of *θ*_A_ the power of H12 should be higher. We modeled incomplete sweeps with *PF* = 0.1, 0.5, and 0.9, with varying times since selection had ceased of *T*_*E*_ = 0, 0.001, and 0.01 in units of 4*N*_*e*_ generations. We simulated sweeps under three selection coefficients, *s* = 0.001, 0.01, and 0.1.

**Fig 6:**
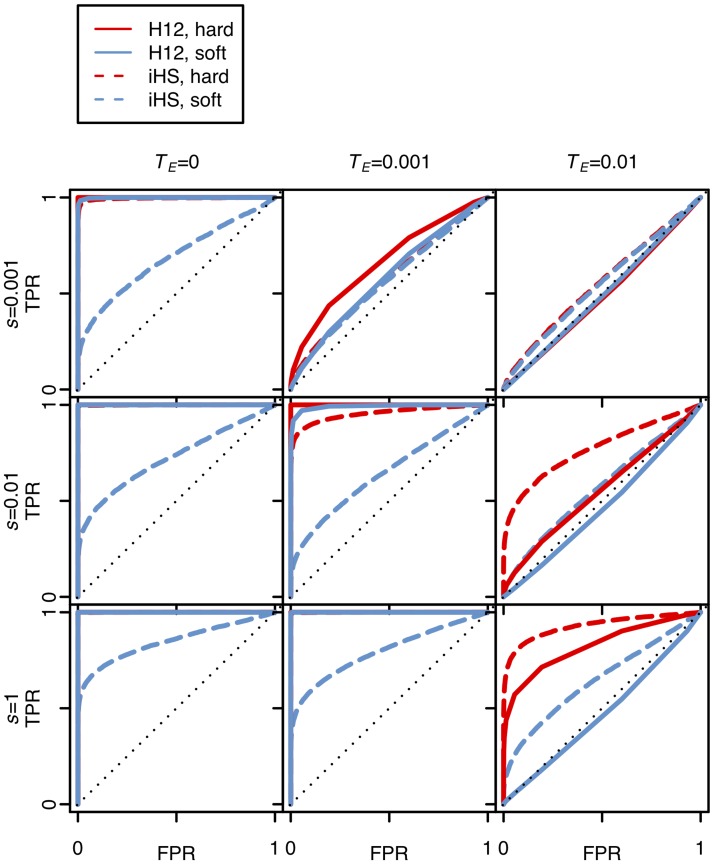
Power analysis of H12 and *iHS* under different sweep scenarios. The plots show ROC curves for H12 and *iHS* under various sweep scenarios with the specified selection coefficients (*s*), and the time of the end of selection (*T*_*E*_) in units of 4*N*_*e*_ generations. In all scenarios, the ending partial frequency of the adaptive allele was 0.5. False positive rates (FPR) were calculated by counting the number of neutral simulations that were misclassified as sweeps under a specific cutoff. True positive rates (TPR) were calculated by counting the number of simulations correctly identified as sweeps under the same cutoff. Hard and soft sweeps were simulated from *de novo* mutations with *θ*_A_ = 0.01 and 10, respectively, under a constant effective population size of *N*_*e*_ = 10^6^, a neutral mutation rate of 10^–9^ bp/gen, and a recombination rate of 5×10^–7^ cM/bp. A total of 5000 simulations were conducted for each evolutionary scenario. H12 performs well in identifying recent and strong selective sweeps, and is more powerful than *iHS* in identifying soft sweeps.

Fig. 6 and S4 Fig. show that the tests based on H12 and *iHS* have similar power for the detection of hard sweeps, although in the case of old and strong hard sweeps (*T*_*E*_ = 0.01, *s* ≥ 0.01) *iHS* performs slightly better than H12. On the other hand, H12 substantially outperforms *iHS* in detecting soft sweeps and has high power when selection is sufficiently strong and the sweeps are sufficiently young. As sweeps become very old, neither statistic can detect them well, as expected.

### H12 scan of DGRP data

We applied the H12 statistic to DGRP data in sliding windows of 400 SNPs with the centers of each window iterated by 50 SNPs. To classify haplotypes within each analysis window, we assigned the 400 SNP haplotypes into groups according to exact sequence identity. If a haplotype with missing data matched multiple haplotypes at all genotyped sites in the analysis window, then the haplotype was randomly assigned to one of these groups (Methods).

**Fig 7:**
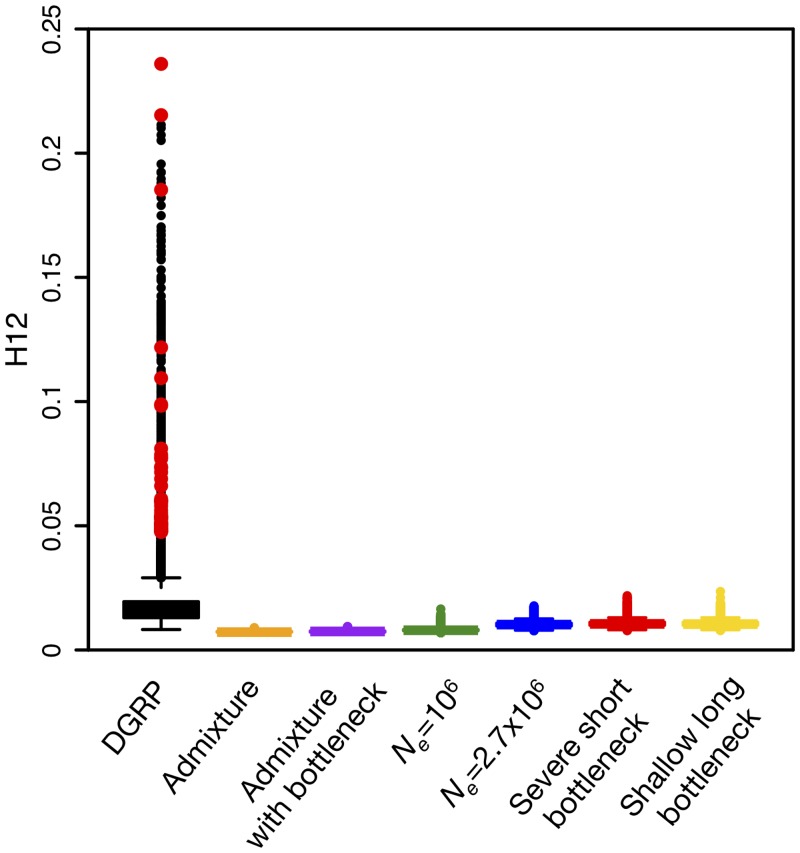
Elevated H12 values and long-range LD in DGRP data. (A) Genome-wide H12 values in DGRP data are elevated as compared to expectations under any neutral demographic model tested. Plotted are H12 values for DGRP data reported in analysis windows with *ρ* ≥ 5×10^-7^ cM/bp. Red dots overlaid on the distribution of H12 values for DGRP data correspond to the highest H12 values in outlier peaks of the DGRP scan at the 50 top peaks depicted in Fig. 8A. Note that most of the points in the tail of the H12 values calculated in DGRP data are part of the top 50 peaks as well. Neutral demographic simulations were generated with *ρ* = 5×10^–7^ cM/bp. Plotted are the result of approximately 1.3x10^5^ simulations under each neutral demographic model, representing ten times the number of analysis windows in DGRP data.

To assess whether the observed H12 values in the DGRP data along the four autosomal arms are unusually high as compared to neutral expectations, we estimated the expected distribution of H12 values under each of the six neutral demographic models. Fig. 7 shows that genome-wide H12 values in DGRP data are substantially elevated as compared to expectations under any of the six neutral demographic models. In addition, there is a long tail of outlier H12 values in the DGRP data suggestive of recent strong selective sweeps.

To identify regions of the genome with H12 values significantly higher than expected under neutrality, we calculated critical values (H12_*o*_) under each of the six neutral models based on a 1-per-genome false discovery rate (FDR) criterion. Our test rejects neutrality in favor of a selective sweep when H12 > H12_*o*_ (Methods and S1 Text). The critical H12_*o*_ values under all neutral demographic models are similar to the median H12 value observed in the DGRP data (Table 1), consistent with the observations of elevated genome-wide haplotype homozygosity and much slower decay in LD at the scale of 10 kb in the DGRP data compared to all neutral expectations (Fig. 2). We focused on the constant *N*_*e*_ = 10^6^ model because it yields a relatively conservative H12_*o*_ value (Table 1) and preserves the most long-range, pair-wise LD in simulations (Fig. 2).

**Table 1:** 1-per-genome FDR critical H12_*o*_ values for different demographic models and recombination rates.

Demographic model	*ρ* = 10^–7^ cM/bp	*ρ* = 5×10^–7^ cM/bp	*ρ* = 10^–6^ cM/bp
Admixture	0.0084	0.0083	0.0083
Admixture and bottleneck	0.0141	0.0092	0.0085
Constant *N*_*e*_ = 10^6^	0.0391	0.0171	0.0126
Constant *N*_*e*_ = 2.7x10^6^	0.0383	0.0168	0.0133
Severe short bottleneck	0.0450	0.0187	0.0131
Shallow long bottleneck	0.0398	0.0181	0.0083

For our genomic scan we chose to use the 1-per-genome FDR value calculated under the constant *N*_*e*_ = 10^6^ model with a recombination rate of 5×10^–7^ cM/bp. Note that most H12_o_ values are similar to the genome-wide median H12 value of 0.0155.

In order to call individual sweeps, we first identified all windows with H12 > H12_*o*_ in the DGRP data set under the constant *N*_*e*_ = 10^6^ model. We then grouped together consecutive windows as belonging to the same ‘peak’ if the H12 values in all of the grouped windows were above H12_*o*_ for a given model and recombination rate (Methods). We then chose the window with the highest H12 value among all windows in a peak and used this H12 value to represent the entire peak.

**Fig 8:**
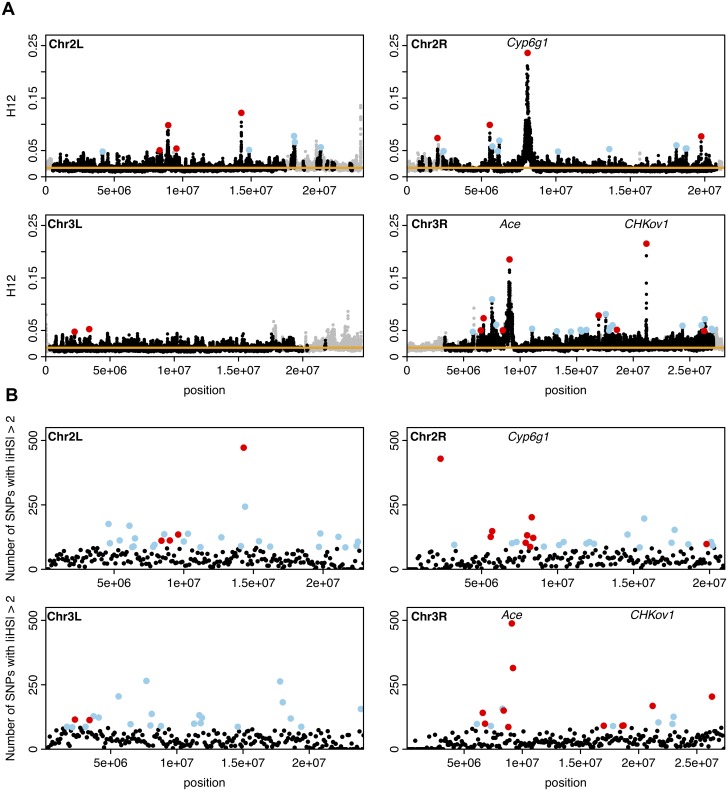
H12 and *iHS* scan in DGRP data along the four autosomal arms. (A) H12 scan. Each data point represents the H12 value calculated over an analysis window of size 400 SNPs centered at the particular genomic position. Grey points indicate regions in the genome with recombination rates lower than 5×10^–7^ cM/bp we excluded from our analysis. The orange line represents the 1-per-genome FDR line calculated under a neutral demographic model with a constant population size of 10^6^ and a recombination rate of 5×10^–7^ cM/bp. Red and blue points highlight the top 50 H12 peaks in the DGRP data relative to the 1-per-genome FDR line. Red points indicate the peaks that overlap the top 10% of 100Kb windows with an enrichment of SNPs with |iHS| > 2 in B. We identify three well-characterized cases of selection in *D*. *melanogaster* at *Ace*, *CHKov1*, and *Cyp6g1* as the three highest peaks. (B) *iHS* scan. Plotted are the number of SNPs in 100Kb windows with |*iHS*| > 2. Highlighted in red and blue are the top 10%100Kb windows (a total of 95 windows). Red points correspond to those windows that overlap the top 50 peaks in the H12 scan. The positive controls, *Ace*, *CHKov1*, and *Cyp6g1* are all among the top 10% windows.

We focused on the top 50 peaks with empirically most extreme H12 values, hypothesized to correspond to the strongest and/or most recent selective events (Fig. 8A). The windows with the highest H12 values for each of the top 50 peaks are highlighted in Fig. 8A. The highest H12 values for the top 50 peaks are in the tail of the distribution of H12 values in the DGRP data (Fig. 7) and thus are outliers both compared to the neutral expectations under all six demographic models and the empirical genomic distribution of H12 values. We observed peaks that have H12 values higher than H12_*o*_ on all chromosomes, but found that there are significantly fewer peaks on 3L (2 peaks) than the approximately 13 out of 50 top peaks expected when assuming a uniform distribution of the top 50 peaks genome-wide (*p* = 0.00016, two-sided binomial test, Bonferroni corrected).

**Fig 9:**
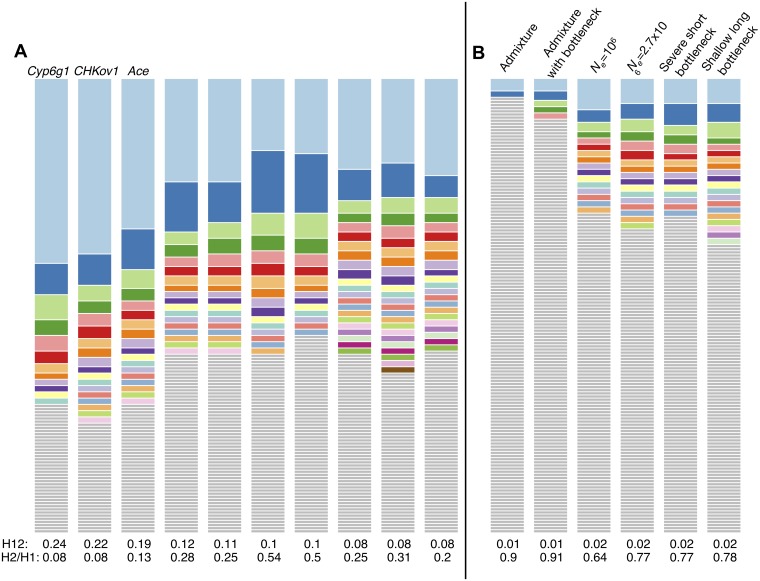
Haplotype frequency spectra for the top 10 peaks and extreme outliers under neutral demographic scenarios. (A) Haplotype frequency spectra for the top 10 peaks in the DGRP scan with H12 values ranging from highest to lowest. For each peak, the frequency spectrum corresponding to the analysis window with the highest H12 value is plotted, which should be the “hardest” part of any given peak. At all peaks there are multiple haplotypes present at high frequency, compatible with signatures of soft sweeps shown in Fig. 5. None of the cases have a single haplotype present at high frequency, as would be expected for a hard sweep. (B) In contrast, the haplotype frequency spectra corresponding to the extreme outliers under the six neutral demographic scenarios have critical H12_*0*_ values that are significantly lower than the H12 values at the top 10 peaks.

The three peaks with the highest observed H12 values correspond to the three known cases of positive selection in *D*. *melanogaster* at the genes *Ace*, *Cyp6g1*, and *CHKov1* [17,19,21], confirming that the H12 scan is capable of identifying previously known cases of adaptation. In S4 Table, we list all genes that overlap with any of the top 50 peaks. Fig. 9A and S5 Fig. show the haplotype frequency spectra observed at the top 50 peaks. In contrast, Fig. 9B shows the frequency spectra observed under the six demographic models with the corresponding critical H12_*o*_ values.

We performed several tests to ensure the robustness of the H12 peaks to potential artifacts (S1 Text). We first tested for associations of H12 peaks with inversions in the sample, but did not find any (S1 Text, S5 Table). In addition, we reran the scan in three different data sets of the same population and confirmed that unaccounted population substructure and variability in sequencing quality do not confound our results (S1 Text, S7 Fig.). We also sub-sampled the DGRP data set to 40 strains ten times and plotted the resulting distributions of H12 values. We found that in all subsamples there is an elevation in haplotype homozygosity relative to neutral demographic scenarios, suggesting that the elevation in haplotype homozygosity values is driven by the whole sample and not a particular subset of individuals (S8 Fig.). Finally, to ensure that haplotype homozygosity is not elevated by family structure, we excluded all related individuals and reran the scan, again recovering the majority of our top peaks (S1 Text, S7 Fig.).

We scanned chromosome 3R using H1 and H123 as our test statistics in order to determine the impact of our choice of grouping the two most frequent haplotypes together in our H12 test statistic on the location of the identified peaks (S9 Fig.). We found that the locations of the identified peaks are similar with all three statistics, but that some smaller peaks that cannot be easily identified with H1 are clearly identified with H12 and H123, as expected.

### *iHS* scan of DGRP data

We applied the *iHS* statistic as described in Voight *et al*. 2006 [40] to all SNPs in the DGRP data to determine the concordance in the sweep candidates identified by *iHS* and H12 (Methods). Briefly, we searched for 100 kb windows that have an unusually large number of SNPs with standardized *iHS* values (|*iHS*|) > 2. The positive controls *Ace*, *Cyp6g1*, and *CHKov1* are located within the 95 top 10% iHS 100 kb windows (Fig. 8B), validating this approach.

To determine how often a candidate region identified in the H12 scan is identified in the *iHS* scan and vice versa, we overlapped the top 50 H12 peaks with the 95 top 10% iHS 100Kb windows. We defined an overlap as the non-empty intersection of the two genomic regions defining the boundaries of a peak in the H12 scan and the non-overlapping 100Kb windows used to calculate enrichment of |*iHS*| values. We found that 18 H12 peaks overlap 28 |*iHS*| 100Kb enrichment windows. In contrast, fewer than 5 H12 peaks are expected to overlap approximately 7 iHS 100Kb windows by chance (Methods). The concordance between the two scans confirms that many of the peaks identified in the two scans are likely true selective sweeps and also suggests that the two approaches are not entirely redundant.

### Distinguishing hard and soft sweeps based on the statistic H2/H1

Our analysis of H12 haplotype homozygosity and the decay in long range LD in DGRP data suggests that extreme outliers in the H12 DGRP scan are in locations of the genome that may have experienced recent and strong selective sweeps. The visual inspection of the haplotype spectra of the top 10 peaks in Fig. 9A and the remaining 40 peaks in S5 Fig. reveals that they contain many haplotypes at substantial frequency. These spectra do not appear similar to those generated by hard sweeps in Fig. 3 or extreme outliers under neutrality in Fig. 9B, but instead visually resemble incomplete soft sweeps with *s* = 0.01 and *PF* = 0.5 either from *de novo* mutations with *θ*_A_ between 1 and 20 or from SGV starting at partial frequencies of 5x10^–5^ to 5x10^–4^ prior to the onset of selection (Fig. 3). The sweeps also appear to become softer as H12 decreases, consistent with our expectation that H12 should lose power for softer sweeps.

In order to gain intuition about whether the haplotype spectra for the top 50 peaks can be more easily generated either by hard or soft sweeps under various evolutionary scenarios, we developed a new haplotype homozygosity statistic, H2/H1, where H2 = Σ_*i*>1_ *p*_*i*_^2^ = H1—*p*_1_^2^ is haplotype homozygosity calculated using all but the most frequent haplotype (Fig. 4C). We expect H2 to be lower for hard sweeps than for soft sweeps because in a hard sweep only one adaptive haplotype is expected to be at very high frequency [53]. The exclusion of the most common haplotype should therefore reduce haplotype homozygosity precipitously. As sweeps get softer, however, multiple haplotypes start appearing at high frequency in the population and the exclusion of the most frequent haplotype should not decrease the haplotype homozygosity to the same extent. Conversely H1, the homozygosity calculated using all haplotypes, is expected to be higher for a hard sweep than for a soft sweep as we described above. The ratio H2/H1 between the two measures should thus increase monotonically as a sweep becomes softer, thereby offering a summary statistic that, in combination with H12, can be used to test whether the observed haplotype patterns are more likely to be generated by hard or soft sweeps. Note that we intend H2/H1 to be measured near the center of the sweep where H12 is the highest. Otherwise, when H2/H1 is estimated further away from the sweep center, mutation and recombination events will decay the haplotype signature and hard and soft sweep signatures can become indistinguishable.

### Softness of sweeps at the top 50 H12 peaks

**Fig 10:**
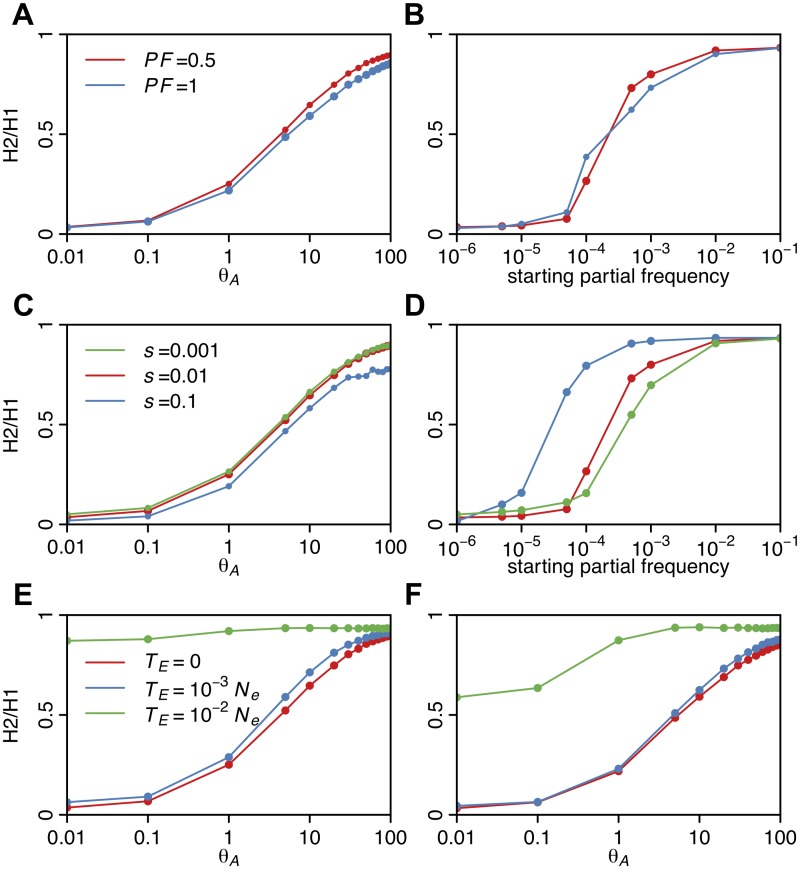
H2/H1 values measured in sweeps of varying softness. Similar to Fig. 5, H2/H1 values were measured in simulated sweeps arising from (A) *de novo* mutations with *θ*_A_ values ranging from 10^–2^ to 10^2^ and (B) SGV with starting frequencies ranging from 10^–6^ to 10^–1^. Sweeps were simulated under a constant *N*_*e*_ = 10^6^ demographic model with a recombination rate of 5×10^-7^ cM/bp, selection strength of *s* = 0.01, ending partial frequencies of the adaptive allele after selection ceased, *PF* = 1 and 0.5, and in samples of 145 individuals. Each data point was averaged over 1000 simulations. H2/H1 values rapidly increase with increasing softness of a sweep, but do not depend strongly on *PF*. In (C) and (D), *s* was varied while keeping *PF* constant at 0.5 for sweeps from *de novo* mutations and SGV, respectively. In the case of sweeps from SGV, H2/H1 values increase as *s* increases, reflecting a hardening of sweeps with smaller *s*. In (E) and (F), the time since selection ended (*T*_*E*_) was varied for incomplete (*PF* = 0.5) and complete (*PF* = 1) sweeps respectively while keeping *s* constant at 0.01. As the age of a sweep increases, the sweep signature decays and H2/H1 approaches one.

To assess the behavior of H2/H1 as a function of the softness of a sweep, we measured H2/H1 in simulated sweeps of varying softness arising from *de novo* mutations and SGV with various *s*, *PF*, and *T*_*E*_ values. Fig. 10 shows that H2/H1 has low values for sweeps with *θ*_A_ ≤ 0.5 or when the starting partial frequency of the adaptive allele prior to the onset of selection is <10^–5^, i.e., when sweeps are mainly hard. As a sweep becomes softer, H2/H1 values approach one because no single haplotype dominates the haplotype spectrum. In the case of sweeps arising from *de novo* mutations, H2/H1 values are similar for partial (*PF* = 0.5) and complete sweeps (*PF* = 1) and for sweeps of varying strengths (*s* = 0.001, 0.01, 0.1). However, in the case of sweeps arising from SGV, sweeps with higher selection strengths do have higher H2/H1 values, reflecting the hardening of sweeps for smaller *s* values as we discussed previously (Fig. 5D). Both sweeps from *de novo* mutations and SGV have higher H2/H1 values for older sweeps, reflecting the decay of the haplotype frequency spectrum over time.

**Fig 11:**
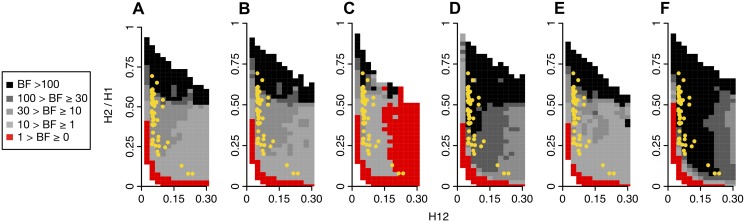
Range of H12 and H2/H1 values expected for hard and soft sweeps. Bayes factors (BFs) were calculated for a grid of H12 and H2/H1 values to demonstrate the range of H12 and H2/H1 values expected under hard versus soft sweeps. Each panel shows the results for a specific evolutionary scenario defined by the underlying demographic model, the *θ*_A_ value used for simulating soft sweeps, and the recombination rate as specified below. BFs were calculated by taking the ratio of the number of soft sweep versus hard sweep simulations that were within a Euclidean distance of 10% of a given pair of H12 and H2/H1 values. Red portions of the grid represent H12 and H2/H1 values that are more easily generated by hard sweeps, while grey portions represent regions of space more easily generated under soft sweeps. Each panel presents the results from one million hard and soft sweep simulations. Hard sweeps were always generated with *θ*_A_ = 0.01. (A), (B), and (C) compare the range of BFs obtained when soft sweeps are generated under *θ*_A_ = 5, 10, and 50, keeping the recombination rate (*ρ*) constant at 5×10^–7^ cM/bp. (A), (D), and (E) compare the range of BFs obtained when *ρ* is varied from 5×10^–7^, 10^–7^, and 10^–6^, keeping the *θ*_A_ constant at 10. (A) and (F) compare the range of BFs generated under the constant *N*_*e*_ = 10^6^ and admixture demographic models for *θ*_A_ = 10 and *ρ* = 5×10^–7^ cM/bp. When H12 values are smaller than 0.05, there is little evidence for a sweep, and most BFs are smaller than one. As H12 values become larger, virtually all sweeps with H2/H1 values > 0.05 are soft. The H12 and H2/H1 values for the top 50 peaks in the DGRP scan are overlaid in yellow. All sweep candidates have H12 and H2/H1 values that are more easily generated by soft sweeps than hard sweeps in most scenarios. (A) Soft sweeps simulated with *θ*_A_ = 10, *ρ* = 5×10^–7^ cM/bp, and a constant *N*_*e*_ = 10^6^ demographic model. (B) Soft sweeps simulated with *θ*_A_ = 5, *ρ* = 5×10^–7^ cM/bp and a constant *N*_*e*_ = 10^6^ demographic model. (C) Soft sweeps simulated with *θ*_A_ = 50, *ρ* = 5×10^–7^ cM/bp, and a constant *N*_*e*_ = 10^6^ demographic model. (D) Soft sweeps simulated with *θ*_A_ = 10, *ρ* = 10^–7^ cM/bp, and a constant *N*_*e*_ = 10^6^ demographic model. (E) Soft sweeps simulated with *θ*_A_ = 10, *ρ* = 10^–6^ cM/bp, and a constant *N*_*e*_ = 10 demographic model. (F) Soft sweeps simulated with *θ*_A_ = 10, *ρ* = 5×10^–7^ cM/bp, and an admixture demographic model.

While hard sweeps and neutrality cannot easily generate both high H12 and H2/H1 values, soft sweeps can do both. In Fig. 11 we assess the range of H12 and H2/H1 values expected under hard and soft sweeps. To compare the likelihood of a hard versus soft sweep generating a particular pair of H12 and H2/H1 values, we calculated Bayes factors: BF = P(H12_obs_, H2_obs_ /H1_obs_ |Soft Sweep)/P(H12_obs_, H2_obs_ /H1_obs_ |Hard Sweep). We approximated BFs using an approximate Bayesian computation (ABC) approach under which the nuisance parameters—selection coefficient (*s*), partial frequency of the adaptive allele after selection has ceased (*PF*), and age (*T*_*E*_)—are integrated out by drawing them from uniform prior distributions: *s* ~ U[0,1], *PF* ~ U[0,1], and *T*_*E*_ ~ U[0,0.001]×4*N*_*e*_. We stated the hard and soft sweep scenarios as point hypotheses in terms of the *θ*_A_ value generating the data. Specifically, we assumed that hard sweeps are generated under *θ*_A_ = 0.01. For soft sweeps, we generated sweeps of varying softness by using *θ*_A_ values of 5, 10, and 50. Note that hard and soft sweeps can also be simulated from SGV with various starting frequencies of the beneficial allele, but for the purposes of generating hard sweeps with a single sweeping haplotype versus soft sweeps with multiple sweeping haplotypes, simulations from SGV or *de novo* mutations are mostly equivalent.

The panels in Fig. 11 show BFs calculated under several evolutionary scenarios for a grid of H12 and H2/H1 values. All panels in Fig. 11 show that hard sweeps are common when H2/H1 values are low for most H12 values tested. For very low H12 (<0.05) values, when sweeps display low haplotype homozygosity to begin with and are difficult to detect with H12, both hard and soft sweeps are likely for a wide range of H2/H1 values. Soft sweeps are common for any high H2/H1 values conditional on H12 being sufficiently high when simulating soft sweeps with *θ*_A_ = 10 and 5 (Fig. 11A and 11B). However, soft sweeps generated with *θ*_A_ = 50 are too soft to produce high H12 values, confirming our results in Fig. 5. As a consequence hard sweeps are common for high H12 values regardless of H2/H1 values under this scenario (Fig. 11C). In Fig. 11A, 11D and 11E, the recombination rate is varied, and a comparison of these panels show that the recombination rate has little impact on the space where hard sweeps can be expected to be more likely. Fig. 11F shows that simulations under admixture increase support for soft sweeps in regions of the space already in support of soft sweeps generated under the constant *N*_*e*_ = 10^6^ demographic scenario (Fig. 11A–E). Fig. 10 shows that there is clearly a dependency between H12 and H2/H1 and that both values need to be taken into account when determining the softness of a peak. In particular, H2/H1 is most informative when applied to regions of the genome with the highest H12 values.

Overlaid on all panels in Fig. 11 are the H12 and H2/H1 values at the top 50 peaks. Note that in almost all cases, the top 50 peaks have H12 and H2/H1 values that are easiest explained by soft sweeps. In order to more explicitly test each candidate sweep for its compatibility with a hard and soft sweep model, we generated hard sweeps with *θ*_A_ = 0.01 and soft sweeps with a maximum a posteriori *θ*_A_ value (*θ*_A_^MAP^), i.e., our best estimate of the softness for a particular peak. We used an ABC method to infer the *θ*_A_^MAP^ for each peak by sampling the posterior distribution of *θ*_A_ conditional on the observed values H12_obs_ and H2_obs_ /H1_obs_ from a candidate sweep (S1 Text). All *θ*_A_^MAP^ values inferred for the top 50 peaks were significantly greater than 1 with the smallest being 6.8 (S10 Fig.), suggesting that soft sweeps would be commonly generated under any of the *θ*_A_^MAP^ values estimated (Fig. 3). We used recombination rates estimated for each peak [49] and simulated the data under the constant population size model with *N*_*e*_ = 10^6^ for computational feasibility. Among our top 50 peaks, we found strong evidence in support of soft sweeps in all 50 cases (BF > 10), very strong evidence in 47 cases (BF > 30), and almost decisive evidence (BF > 98) in 44 cases (S3 Table). Taken together, these results provide evidence that soft sweeps most easily explain the signatures of multiple haplotypes at high frequency observed at the top 50 H12 peaks.

## Discussion

In this study, we found compelling evidence for a substantial number of recent and strong selective sweeps in the North Carolina population of *D*. *melanogaster* and further found that practically all these events appear to display signatures of soft rather than hard sweeps. To detect sweeps, we used our new haplotype statistic, H12, which measures haplotype homozygosity after combining the frequencies of the two most abundant haplotypes into a single frequency in windows of 400 SNPs (~10 kb in the DGRP data).

We chose to use windows defined by a constant number of SNPs rather than windows of constant physical or genetic length in order to simplify the statistical analysis. This is because windows of constant physical or genetic length tend to have varying SNP density, and therefore also varying distributions of haplotypes even under neutrality. Our choice of a fixed number of SNPs avoids this source of noise, but it raises the question of whether the H12 peaks simply define regions that have particularly low recombination rates or high SNP densities, and thus short windows in terms of the number base pairs or genetic map length. We made sure to avoid the first pitfall by analyzing only windows with reasonably high recombination rates (*ρ* ≥ 5x10^–7^ cM/bp, 82% of the genome) and by using conservative thresholds for the significance cutoffs. We also confirmed that the analysis windows with the highest H12 values in our top 50 peaks do not have shorter windows in terms of base pairs than on average (S11 Fig.). We were further concerned that our choice of using windows with a fixed number of SNPs would bias us against detecting complete hard sweeps. However, our simulations showed that this was not the case (Fig. 5).

We fully acknowledge that the result of applying the haplotype statistics developed in this manuscript to the North Carolina population may be idiosyncratic to the particular demographic structure of this one population. However, H12 in the DGRP data is substantially elevated compared to the expectation under any of the tested neutral demographic models, including both published admixture models [45] and the bottleneck models we fit to the DGRP short intron SNP data. In fact, the median value of H12 in the genome lies in the tails of distributions of H12 values generated from > 10^5^ simulations for each neutral demographic scenario. Similarly, pairwise LD in DGRP data decays much more slowly than expected under neutrality (Fig. 2). These patterns can be due either to (i) pervasive and strong positive selection that drives long haplotypes to high frequency in the population, (ii) misspecification of the demographic model, or (iii) both. Although background selection (BGS) is pervasive in *D*. *melanogaster* [55] and strongly impacts levels of polymorphism, it is unlikely to be responsible for high levels of haplotype homozygosity [56,57].

Both selective and neutral demographic explanations of the elevated LD need to be investigated further. It will be important to determine whether current estimates of the rate and strength of adaptation in *D*. *melanogaster* are consistent with the elevated levels of haplotype homozygosity and LD in general, even under simple demographic models. Alternatively, an unusually high rate of adaptation in the recent past might be required to explain the signatures we observe in the data. Likewise, it is possible that some demographic model of the North Carolina population, which is yet to be specified, can account for the observed LD patterns. Both extensive forward simulations and additional studies of LD and haplotype homozygosity patterns in other populations will be important to resolve these issues.

Importantly, however, the top fifty H12 peaks we focused on in this study are outliers not only under all tested demographic models, but also relative to the empirical genome wide H12 distribution. The top three peaks correspond to the well-known cases of soft selective sweeps arising from *de novo* mutations and SGV at the loci *Ace*, *Cyp6g1*, and *CHKov1* [17,19,21] as described in the Introduction. The recovery of these positive controls further validates that our method can identify sweeps arising from both *de novo* mutations and SGV and is robust to misspecifications of demographic models.

In order to confirm the robustness of the H12 peaks, we ran *iHS* [40] on the DGRP data and recovered 18 of the top 50 peaks, including the three positive controls, demonstrating the validity of both methods and that the two methods are not entirely redundant (Fig. 8B). We also failed to detect any correlation between H12 peaks and inversions in the genome. We tested for any unaccounted substructure in the data confounding our results by rerunning the scan in several data sets, including one where all related individuals were excluded. In all cases, we found that our top peaks remained unchanged and that haplotype homozygosity was consistently elevated in the data relative to neutral demographic simulations (S1 Text). We are thus confident that the top H12 peaks are true outliers and likely indicate recent and strong selective events in the North Carolina population of *D*. *melanogaster*.

To assess whether the top 50 peaks can be more easily generated by hard versus soft sweeps, we developed a second statistic, H2/H1, which is a ratio of haplotype homozygosities calculated without (H2) and with (H1) the most frequent haplotype in a sample. We demonstrate that this statistic has a monotonically increasing relationship with the softness of a sweep (Fig. 10), in contrast to H12, which has a monotonically decreasing relationship with the softness of a sweep.

H2/H1 and H12 together are informative in determining the softness of a sweep. Specifically, hard sweeps can generate high values of H12 in a window centered on the adaptive site but cannot simultaneously generate high H2/H1 values in the same window. However, soft sweeps can generate both high H12 and H2/H1 values in such a window. Note that in order to differentiate hard and soft sweeps with reasonable power, H2/H1 can only be applied in cases where H12 values are already high and there is strong evidence for a sweep. Indeed, as can be seen in all evolutionary scenarios presented in Fig. 11, when H12 is high and H2/H1 is low, hard sweeps are common, and when both H12 and H2/H1 are high, soft sweeps are common. However, when H12 is low, *i*.*e*. when there is little evidence for a sweep to begin with, either because the sweep was driven by weak selection or happened a long time ago, a wider range of H2/H1 values are compatible with hard sweeps. This demonstrates that H2/H1 can be used only in windows with very high H12 values. In most cases this should not unduly restrict the analysis as all robustly identified sweeps must have high H12 values given the difficulties of correctly specifying demographic models for any population.

The visual inspection (Fig. 9 and S5 Fig.) and the Bayesian analysis of the H12 and H2/H1 values suggest that all top 50 H12 peaks were driven by soft sweeps. Note that we simulated hard and soft sweeps for the Bayesian analysis under the constant *N*_*e*_ = 10^6^ demographic model for computational feasibility and to make our analysis conservative for the purposes of rejecting the hard sweep scenario. This is because the lower SNP density in the *N*_*e*_ = 10^6^ model (S3 Table), as compared to DGRP data, effectively increases the analysis window size in terms of base pairs, and by extension, also increases the number of recombination events each window experiences. Thus, hard sweeps should look “softer” under this choice of demographic model [53]. Even still, soft sweeps and not hard sweeps seem to more easily explain the signatures at our top 50 peaks.

If soft sweeps are indeed common in *D*. *melanogaster*, then adaptation must commonly act on SGV at low enough frequencies to generate high H12 values or involve multiple *de novo* adaptive mutations entering the population simultaneously. The SGV scenario is clearly plausible, particularly if much adaptation in out-of-Africa populations of *D*. *melanogaster* utilized variants that are rare in Africa. We do, however, expect that many adaptive events will involve SGV at higher frequencies and such adaptive events will generate sweeps that are too soft to be detectable using the H12 statistic. Similarly, *θ*_A_ values much larger than 10 will also generate sweeps too soft to be detected by H12. Curiously, this upper bound of *θ*_A_ is consistent with the median *θ*_A_ inferred from our top 50 peaks, ~12.8 (S10 Fig.). This coincidence suggests that we might still be missing many sweeps that are too soft for detection using H12.

Is it plausible that some of the sweeps were generated by *de novo* mutation? The answer must be clearly yes given that two of three known cases of recent adaptation, at *Ace* and *Cyp6g1*, were generated by *de novo* mutation. In order for this to be possible, the total population scaled adaptive mutation rate (*θ*_A_) must be on the order of one or even larger [27,29]. The commonly assumed value of *N*_*e*_ = 10^6^ for the effective population size in *D*. *melanogaster* and mutation rate per base pair (~10^–9^ bp/generation [48]) implies *θ*_A_ values of approximately 1%, assuming that adaptation at a given locus relies on mutation at a single nucleotide. One reason why *θ*_A_ can be commonly greater than 0.01 is that many mutations at a locus can be adaptive, for instance if adaptation relies on gene loss and any stop codon or indel is equally adaptive. In this case, all such adaptive mutations at a locus will combine to generate a soft sweep.

In addition, the population size relevant for recent adaptation might be much closer to the census population size at the time of adaptation and thus can be much larger than the commonly assumed value of *N*_*e*_ = 10^6^ for the effective population size in *D*. *melanogaster*. We favor this explanation of a much larger effective population size of *D*. *melanogaster* relevant for recent and strong adaptation for two reasons. First, it is unlikely that every single case of recent and strong adaptation was driven by a situation where the adaptive mutation rate at a locus was a hundred times higher than a mutation rate at a single site. Second, in the case of adaptation at *Ace*, adaptation was driven by three point mutations, and the soft sweeps at *Ace* are incompatible with the relevant population size being on the order of 10^6^ [17]. The relevant population size for recent and strong adaptation in *D*. *melanogaster* should be thus more than 100-fold than 10^6^. Note that the relevant population size here is that of the *D*. *melanogaster* population as a whole and not just the North Carolina DGRP population. A likely possibility is that we observe signatures of multiple local hard sweeps arising within sub-demes of the North American Drosophila population or in the ancestral European and African populations prior to admixture, that combine to generate signatures of soft sweeps [58].

Nevertheless, it is quite puzzling that we were unable to detect any hard sweeps. One possibility is that hard sweeps do exist but are driven by weaker selection than we can detect in our scan. Indeed, Wilson *et al*. [52] argued that sweeps driven by weak selection could become hard even when they occur in populations of large size. This is because such sweeps take a long enough time to increase in frequency allowing rare but sharp bottlenecks to eliminate all but the highest frequency adaptive allele. It is also possible that hard sweeps were common in the past and degraded over time, while recent adaptation from *de novo* or rare variants produced primarily soft sweeps. While it is possible that hard sweeps correspond to the weaker and older selection events that we lack the power to identify, it is reassuring that our method is biased toward discovering the strongest, most recent, and thus most consequential adaptive events in the genome.

The abundance of signatures of soft sweeps in *D*. *melanogaster* has important implications for the design of methods used to quantify adaptation. Some methods may work equally well whether adaptation proceeds via hard or soft sweeps. For instance, estimates of the rate of adaptive fixation derived from McDonald-Kreitman tests [59] are not expected to be affected strongly because these estimates depend on the rate of fixation of adaptive mutations and not on the haplotype patterns of diversity that these adaptive fixations generate in their wake. Tests based on the prediction that regions of higher functional divergence should harbor less neutral diversity [10,11,60] are generally consistent with recurrent hard and soft sweeps, as both scenarios are expected to increase levels of genetic draft, and thus reduce neutral diversity in regions of frequent and recurrent adaptation. Note that soft sweeps generate less of a reduction in neutral diversity. As a consequence, such methods might underestimate the rate of adaptation. However, methods that quantify adaptation based on a specific functional form of the dependence between the level of functional divergence and neutral diversity may lead to different conclusions under hard and soft sweeps [10]. Finally, methods that rely on the specific signatures of hard sweeps, such as the presence of a single frequent haplotype [39,40], sharp local dips in diversity [22], or specific allele frequency spectra expected during the recovery after the sweep might often fail to identify soft sweeps [35]. Hence, such methods might give us an incomplete picture of adaptation. Moreover, such methods might erroneously conclude that certain genomic regions lacked recent selective sweeps, which can be problematic for demographic studies that rely on neutral polymorphism data unaffected by linked selection.

Our statistical test based on H12 to identify both hard and soft sweeps and our test based on H12 and H2/H1 to distinguish signatures of hard versus soft sweeps can be applied in all species in which genome-scale polymorphism data are available. The current implementation requires phased data but the method can easily be extended to unphased data as well by focusing on the frequencies of homozygous genotypes. Our method requires a sufficiently deep population sample for the precise measurement of haplotype frequencies, which is essential for determining whether a haplotype is unusually frequent in the sample. For example, in our DGRP scan, the majority of the 50 highest H12 peaks had a combined frequency of the two most common haplotypes below 30%, while only the top three peaks had a combined frequency of approximately 45%. Determination of whether a sweep is hard or soft should be particularly sensitive to the depth of the population sample. Finally, in order to determine whether an observed H12 value is sufficiently high enough to suggest that a sweep has occurred in the first place, reliable estimates of recombination rates are needed. We encourage the use of an empirical outlier approach to identify sweep candidates, especially because it is often difficult to accurately infer appropriate demographic models.

Our results provide evidence that signatures of soft selective sweeps were abundant in recent evolution of *D*. *melanogaster*. Soft sweep signatures may be common in many additional organisms with high census population sizes, including plants, marine invertebrates, insects, microorganisms, and even modern humans when considering very recent evolution in the population as a whole. Indeed, the list of known soft sweeps is large, phylogenetically diverse, and is constantly growing [14]. A comprehensive understanding of adaptation therefore must account for the possibility that soft selective sweeps are a frequent and possibly dominant mode of adaptation in nature.

## Methods

### Simulations of selection and neutrality

Population samples under selection and neutrality were simulated with the coalescent simulator MSMS [61]. We simulated samples of size 145 to match the sample depth of the DGRP data and always assumed a neutral mutation rate of 10^–9^ events/bp/gen [48].

MSMS can simulate selective sweeps both from *de novo* mutations and SGV. We simulated sweeps of varying softness arising from *de novo* mutations by specifying the population parameter *θ*_A_ = 4*N*_*e*_μ_A_ at the adaptive site. We simulated sweeps arising from SGV by specifying the initial frequency of the adaptive allele in the population at the onset of positive selection. The adaptive site was always placed in the center of the locus. We assumed co-dominance, whereby a homozygous individual bearing two copies of the advantageous allele has twice the fitness advantage of a heterozygote. To simulate incomplete sweeps we specified the ending partial frequency of the adaptive allele after selection has ceased. To simulate sweeps of different ages, we conditioned on the ending time of selection (*T*_*E*_) prior to sampling.

When simulating selection with the admixture demographic model, it was unfortunately not possible in MSMS to condition on *T*_*E*_. For this demographic scenario, we instead conditioned on the start time of selection in the past and the starting partial frequency of the adaptive allele prior to the onset of selection, with selection continued until the time of sampling. In doing so, we assumed a uniform prior distribution of the start time of selection, U[0 to 3.05×10^–4^ *N*_*e*_] generations, with the upper bound specifying the time of the admixture event.

### Performance analysis of haplotype statistics

We simulated loci of length 10^5^ bp for sweep simulations with *s* < 0.1 and 10^6^ bp for sweep simulations with *s* = 0.1. For neutral simulations, we simulated loci of length 10^5^ bp. We assumed a constant effective population size of *N*_*e*_ = 10^6^ and a recombination rate of 5×10^–7^ cM/bp, reflecting the cutoff used in the DGRP analysis.

Our statistics H12 and H2/H1 were estimated over windows of size 400 SNPs centered on the adaptive site. Simulated samples that yielded fewer than 400 SNPs were discarded. For the comparison with *iHS*, we calculated *iHS* values for the SNP immediately to the right of the selected allele, and determined the size of the region by cut-off points at which *iHS* levels decayed to values observed under neutrality. In some simulation runs under the extreme scenario with *s* = 0.1 and *T*_*E*_ = 0, *iHS* had not yet decayed to neutral levels at the edges of the simulated sweep. However, this should have only minor impact on the ROC curves.

### Quality filtering of the DGRP data

The DGRP data set generated by Mackay *et al*. (2012) [44] consists of the fully sequenced genomes of 192 inbred *D*. *melanogaster* lines collected from Raleigh, North Carolina. Reference genomes are available only for 162 lines. Of these 162 lines, we filtered out a further 10% of the lines with the highest number of heterozygous sites in their genomes, possibly reflecting incomplete inbreeding. The IDs of these strains are: 49, 85, 101, 109, 136, 153, 237, 309, 317, 325, 338, 352, 377, 386, 426, 563, and 802. Any remaining residual heterozygosity in the data was treated as missing data. Our final data set consisted of 145 strains.

### Linkage disequilibrium estimates

We measured linkage disequilibrium (LD) in DGRP data and in simulations of neutral demographic scenarios in samples of size 145. Simulations were performed assuming a neutral mutation rate of 10^–9^ events/bp/gen and a recombination rate of 5x10^–9^ cM/bp. LD was measured using the R^2^ statistic in sliding windows of 10 kb iterated by 50 bps. LD was measured between the first SNP in the window with an allele frequency between 0.05 and 0.95 and the rest of the SNPs in the window with allele frequencies between 0.05 and 0.95. If any SNP had missing data, the individuals with the missing data were excluded from the LD calculation. At least 4 individuals without missing data at both SNPs were required to compute LD, otherwise the SNP pair was discarded. LD plots were smoothed by averaging LD values binned in non-overlapping 20 bp windows until a distance of 300 bps. After that, LD values were averaged in bins of 150 bp non-overlapping windows.

### Genomic scan for selective sweeps in DGRP using H12

We scanned the genome using sliding windows of 400 SNPs with intervals of 50 SNPs between window centers and calculated H12 in each window. If two haplotypes differed only at sites with missing data, we clustered these haplotypes together. If multiple haplotypes matched a haplotype with missing data, we clustered the haplotype with missing data at random with equal probability with one of the other matching haplotypes. We treated heterozygous sites in the data as sites with missing data (“N”).

To identify regions with unexpectedly high values of H12 under neutrality, we calculated the expected distribution of H12 values under the admixture, admixture and bottleneck, constant *N*_*e*_ = 10^6^, constant *N*_*e*_ = 2.7x10^6^, severe short bottleneck, and shallow long bottleneck demographic scenarios specified in Fig. 1. For each scenario, we simulated ten times the number of independent analysis windows (approximately 1.3x10^5^ simulations) observed on chromosomes 2L, 2R, 3L, and 3R using three different recombination rates: 10^–7^ cM/bp, 5×10^–7^ cM/bp, and 10^–6^ cM/bp. All simulations were conducted with locus lengths of 10^5^ basepairs. We assigned a 1-per-genome FDR level to be the 10th highest H12 value in each scenario.

Consecutive windows with H12 values that are above the 1-per-genome-FDR level were assigned to the same peak by the following algorithm: first, we identified the analysis window with the highest H12 value along a chromosome above the 1-per-genome-FDR with a recombination rate greater than 5×10^–7^ cM/bp. We then grouped together all consecutive windows with H12 values that lie above the cutoff and assigned all these windows to the same peak. After identifying a peak, we chose the highest H12 value among all windows in the peak to represent the H12 value of the entire peak. We repeated this procedure for the remaining windows until all analysis windows were accounted for.

### Genomic scan of DGRP data with *iHS*

We scanned the DGRP data using a custom implementation of the *iHS* statistic written by Sandeep Venkataram and Yuan Zhu. *iHS* was calculated for every SNP with a minor allele frequency (MAF) of at least 0.05 without polarization. Any strain with missing data in the region of extended haplotype homozygosity for a particular SNP was discarded in the computation of *iHS*. All *iHS* values were standardized by the mean and variance of *iHS* values calculated at all SNPs sharing a similar MAF (within ± 0.05). As described in Voight *et al*. [40], we calculated the enrichment of SNPs with standardized *iHS* values > 2 in non-overlapping 100 Kb windows.

### Expected number of overlapping candidate regions in the H12 and *iHS* scans

To determine the number of top H12 peaks that should overlap the top |*iHS*| enrichment regions by chance, we calculated the expected fraction of the genome that should overlap the top candidates in both scans. The top 50 H12 peaks cover a total of 7,166,386 bps of the genome, or, 7.42% of the genome. Similarly, the top 95 |*iHS*| enrichment windows with |*iHS*| > 2 cover 9,500,000 bps of the genome, or 9.83% of the genome. Thus, only 0.73% of the genome should overlap both the top H12 peaks and top |*iHS*| enrichment windows by chance. Multiplying this percentage with the total number of bps in the DGRP data set (96,595,864) and normalizing by the total area of the genome covered by the top 50 H12 peaks and top 95 |*iHS*| enrichment regions, only ~10% of the fraction of the genome covered by H12 peaks should overlap ~7.4% of the fraction of the genome covered by |*iHS*| enrichment regions. Assuming a uniform distribution of H12 peaks in the region of the genome covered by H12 peaks, approximately 5 H12 peaks should overlap approximately 7 |*iHS*| enrichment regions by chance.

### Demographic inference with DaDi

We fit six simple bottleneck models to DGRP data using a diffusion approximation approach as implemented by the program DaDi [47]. DaDi calculates a log-likelihood of the fit of a model based on an observed site frequency spectrum (SFS).

We estimated the SFS for presumably neutral SNPs in the DGRP using segregating sites in short introns [62]. Specifically, we used every site in a short intron of length less than 86 bps, with 16 bps removed from the intron start and 6 bps removed from the intron end [63]. We projected the SFS for our data set down to 130 chromosomes (after excluding the top 10% of strains with missing data), resulting in 42,679 SNPs out of a total of 738,024 bps.

We specified a constant population size model as well as six bottleneck models with the sizes of the bottlenecks ranging from 0.2% to 40% of the ancestral population size. Using DaDi [47], we inferred three free parameters: the bottleneck time (*T*_*B*_), final population size (*N*_*F*_), and the final population time (*T*_*F*_) (S1 Fig. and S2 Table). All six bottleneck models produced approximately the same log likelihood values and estimates of *N*_*F*_ and *T*_*F*_. Further, the estimates of *S* and *π* obtained from simulated data matched the estimates obtained from the observed short intron data (S3 Table). Note that the estimate of *T*_*B*_ is proportional to *N*_*B*_, reflecting the difficulty in distinguishing short and deep bottlenecks from long and shallow bottlenecks. We inferred *N*_*e*_ = 2,657,111 (≈2.7x10^6^) for the constant population size model, assuming a mutation rate of 10^–9^/bp/generation.

### ABC inference of *θ*_A_^MAP^ for top 50 peaks

To infer *θ*_**A**_^**MAP**^ values for the top 50 peaks (S1 Text), we assumed uniform distributions for all model parameters in our ABC procedure: The adaptive mutation rate (*θ*_A_) took values on [0,100], the selection coefficient *s* on [0,1], the ending partial frequency of the adaptive allele after selection has ceased (*PF*) on [0,1], and the age of the sweep (*T*_*E*_) on [0,0.001]×4*N*_*e*_. We assigned a recombination rate to each peak according to the estimates from Comeron *et al*. (2012) [49] for the specific locus. For the ABC procedure, we binned recombination rates into 5 equally spaced bins. Then, for each peak, we simulated the recombination rate from a uniform distribution over the particular bin its recombination rate fell in. The recombination rate intervals defining the 5 bins were: [5.42*10^–7^, 1.61*10^–6^), [1.61*10^–6^, 2.68*10^–6^), [2.68*10^–6^, 3.74*10^–6^), [3.74*10^–6^, 4.81*10^–6^), [4.81*10^–6^, 5.88*10^–6^) in units of cM/bp. We assumed a demographic model with constant *N*_*e*_ = 10^6^ and a non-adaptive mutation rate of 10^–9^ bp/gen in our simulations.

For each peak, we sampled an approximate posterior distribution of *θ*_A_ by finding 1000 parameter values that generated sweeps with H12 and H2/H1 values within 10% of the observed values H12_obs_ and H2_obs_ /H1_obs_ for the particular peak. We calculated the lower and upper 95% credible interval bounds for *θ*_A_ using the 2.5^th^ and 97.5^th^ percentiles of the posterior sample. On each posterior sample, we applied a Gaussian smoothing kernel density estimation and obtained the maximum a posteriori estimate *θ*_A_^MAP^ for each peak.

We used the same procedure for obtaining approximate posterior distributions of *θ*_A_ and *θ*_A_^MAP^ estimates under the admixture model. In this case, instead of sampling the time when selection ceased, we sampled the time of the onset of selection with uniform prior distribution: U[0, 3.05×10^–4^]×*N*_*e*_, where 3.05×10^–4^ *N*_*e*_ generations is the time of the admixture event. The prior distributions for all other parameters were the same as for the constant *N*_*e*_ = 10^6^ model.

### Test of hard versus soft sweeps for the top 50 peaks

We used an ABC approach to calculate Bayes factors for a range of H12 and H2/H1 values. We simulated hard sweeps with *θ*_A_ = 0.01 and soft sweeps with *θ*_A_ = 5, 10, 50, or the *θ*_A_^MAP^ inferred for a particular peak, depending on the scenario being tested. In the constant *N*_*e*_ = 10^6^ models shown in Fig. 11A–E, selection coefficients, partial frequencies of the adaptive allele after selection has ceased, and sweep ages were drawn from uniform distributions as follows: *s* ~ U[0,1], *T*_*E*_ ~ U[0, 10^4^]×4*N*_*e*_, *PF* ~ U[0,1]. For the admixture model in Fig. 11F, the age of the onset of selection was sampled from a uniform distribution: U[0, 3.05×10^–4^]*N*_*e*_ generations, where 3.05×10^–4^ *N*_*e*_ generations corresponds to the time of the admixture event.

We calculated Bayes factors by taking the ratio of the number of data sets simulated with H12 and H2/H1 values with a Euclidean distance < 0.1 from the observed values H12_obs_ and H2_obs_ /H1_obs_ for each set of 10^6^ simulated data sets under soft versus hard sweeps (10^5^ data sets were generated for explicitly testing each peak with *θ*_A_^MAP^). We calculated the Euclidean distance as follows: *d*_*i*_ = [(H12_obs_—H12_i_)^2^ /Var(H12) + (H2_obs_/H1_obs_—H2_i_/H1_i_)^2^ /Var(H2/H1)]^1/2^, where Var(H12) and Var(H2/H1) are the estimated variances of the statistics H12 and H2/H1 calculated using all simulated data sets.

## Supporting Information

S1 Text Calculation of the 1-per-genome FDR critical value of H12o, robustness of the H12 scan, and estimation of *θ*_A_ for the top 50 peaks. (PDF)

S1 Fig Simple bottleneck models inferred by DaDi. The inferred parameters were the size of the final population (*NF*), the duration of the bottleneck (*TB*), and the time after the bottleneck (*TF*). Investigated bottleneck sizes ranged from *NB* = 0.002 to *NB* = 0.4 (see S2 Table). *NB* = 0.002 represents the population size of the bottleneck inferred for European flies by Li and Stephan (2006) [64], whereas *NB* = 0.4 represents a comparatively shallow population size reduction.
(TIF)

S2 Fig Higher number of haplotypes (*K*) in under the admixture model versus the constant *N*_*e*_ = 10^6^ model. We observe a significantly higher number of unique haplotypes (*K*) in neutral simulations of admixture as compared to a constant *N*_*e*_ scenario. Here we plot distributions of *K* in a sample of haplotypes drawn from the North American deme in the admixture model in Fig. 1 and a constant *N*_*e*_ = 10^6^ model. In each scenario, 1000 simulations were performed.
(TIF)

S3 Fig H1, H12, and H123 values measured in sweeps of varying softness. Homozygosity values were measured in simulated sweeps arising from (A) *de novo* mutations with *θ*_A_ values ranging from 10^–2^ to 10^2^ and (B) SGV with starting frequencies ranging from 10^–6^ to 10^–1^. Sweeps were simulated under a constant *N*_*e*_ = 10^6^ demographic model with a recombination rate of 5×10^–7^ cM/bp, selection coefficient of *s* = 0.01, and ending partial frequency of the adaptive allele after selection ceased, *PF* = 0.5. Each data point was averaged over 1000 simulations. H1, H12, and H123 values all decline rapidly as the softness of a sweep increases. H12 modestly augments our ability to detect a sweep as long as the sweep is not too soft or too old. H123 has marginally better ability to detect selective sweeps as compared to H12.
(TIF)

S4 Fig Power analysis of H12 and *iHS* under different sweep scenarios. Same as Fig. 6, except ending partial frequencies of the adaptive allele after selection ceased are *PF* = 0.1 in (A) and *PF* = 0.9 in (B).
(TIF)

S5 Fig Haplotype frequency spectra for the 11^th^-50^th^ peaks. Same as Fig. 9, except plotted are haplotype frequency spectra for the (A)11^th^-30^th^ and the (B) 31^st^—50^th^ peaks in the DGRP scan.
(TIF)

S6 Fig Elevated H12 values in DGRP data excluding regions overlapping inversions. Similar to Fig. 7, except here regions overlapping major cosmopolitan inversions are excluded from the distribution of H12 values in DGRP data. There is a long tail and elevation of H12 values in DGRP data as compared to expectations under any neutral demographic model tested.
(TIF)

S7 Fig H12 scan in three additional data sets of the North Carolina *D*. *melanogaster* population. We reran the H12 scan in three data sets: (A) DPGP data, (B) DGRP version 2 data set, and (C) the 63 DGRP version 2 strains that do not overlap the 145 strains used in the original DGRP scan. Blue and red points highlight the top 50 most extreme peaks with high H12 values relative to the median H12 value in the scan. Red points indicate peaks among the top 50 in each scan that overlap the top 50 peaks observed in the original DGRP scan. In (A), 16 peaks overlap, in (B), 40 peaks overlap, and in (C), 12 peaks overlap. Most of the overlapping peaks are among the top ranking peaks in the DGRP scan. We identify the three well-characterized cases of selection in *D*. *melanogaster* at *Ace*, *CHKov1*, and *Cyp6g1* in all three scans.
(TIF)

S8 Fig Elevation in H12 values in DGRP data after down sampling to 40 strains. DGRP strains were downsampled to 40 strains 10 times and the resulting distributions of H12 were plotted (black). In contrast to expectations under any neutral demographic model tested with a sample size of 40, all samples of 40 strains have elevated H12 values and a long tail. This indicates that the elevation of homozygosity values observed in DGRP data in Fig. 7 is driven by a population-wide signal and not by any sub-population.
(TIF)

S9 Fig H1, H12, and H123 scan of chromosome 3R. All statistics are able to identify similar peaks. The known cases of adaptation at *Ace* and *CHKov1* have more pronounced peaks under H12 and H123.
(TIF)

S10 Fig Posterior distributions of *θ*_A_ and *θ*_A_^MAP^ estimates for top peaks. (A) Posterior distributions of *θ*_A_ measured under the constant *N*_*e*_ = 10^6^ model and the admixture model (black and grey lines, respectively) and the corresponding *θ*_A_^MAP^ estimates (dashed red and green lines, respectively) for the top nine peaks. (B) Distribution of *θ*_A_^MAP^ values inferred under the constant *N*_*e*_ = 10^6^ model for the top 50 peaks. (C) Corresponding distribution under the admixture model. The distribution of *θ*_A_^MAP^ peaks around *θ*_A_ = 10 under the constant *N*_*e*_ = 10^6^ model and peaks at a slightly higher value under the admixture model, suggesting that the constant *N*_*e*_ = 10^6^ model may be conservative for the purposes of inferring the softness of a sweep.
(TIF)

S11 Fig Distribution of analysis window lengths compared with mean window length for top 50 peaks. To confirm that the the analysis windows with the highest H12 values for our top 50 peaks are not unusually short, we plotted the distribution of window lengths for randomly chosen analysis windows genome-wide. For each of the top 50 peaks, 500 analysis windows with recombination rates within 10% of the observed recombination rate in analysis window with the highest H12 values were drawn randomly. A total of 25,000 windows comprise the distribution below. Plotted in red is the mean window length of the analysis windows for the top 50 peaks. The left tail empirical *P*-value is 0.77.
(TIF)

S1 Table Parameter values used for simulations of admixture models from Fig. 1. Point estimates were calculated by Pablo Duchen (personal communication). All population sizes are in units of *N*_*Ac*._ In the admixture model (A), *N*_*Ac*_ = 4,975,360, and in the admixture with bottleneck model (B), *N*_*Aa*_ = 3,100,520. All times are in units 4*N*_*Ac*_.
(PDF)

S2 Table Demographic parameters inferred by DaDi for simple bottleneck scenarios. Shown are parameter estimates for six simple bottleneck scenarios fit to short intron data in DGRP inferred by DaDi [47] and the corresponding log likelihoods for each model (LL). For all inferred models, the bottleneck sizes (*N*_*B*_) were fixed at values as specified in the table. All population size estimates are in terms of units 4*Ne_ancestral_, and all time estimates are in terms of units 2*Ne_ancestral_. Values of *θ*_exp_ were measured for each inferred demographic models and are a function of the number of base pairs (738,024) used to generate the SFS. Note that *N*_*B*_ = 0.002 represents the population size of the bottleneck inferred by Li and Stephan (2006) [64] and *N*_*B*_ = 0.029 is the population size of the bottleneck inferred by Thornton and Andolfatto (2006) [46]. We ultimately chose to use the short severe bottleneck model (*N*_*B*_ = 0.002, *T*_*B*_ = 0.0002) and shallow long bottleneck model (*N*_*B*_ = 0.4, *T*_*B*_ = 0.0560) because all models fit the data equally well and these two models represent the extreme ends of the range of models tested. See S3 Table for a comparison of the fit of the severe short and shallow long bottleneck models to short intron data in terms of *S* and *π*.
(PDF)

S3 Table *S* and *π* measured in neutral demographic models of North American Drosophila. Estimates of *S* and *π* were averaged over 30,000 simulations of 10,000 bps for each demographic model. *S* and *π* estimates in DGRP short intron data were measured to be 5.8% and 1.2% per bp, respectively.
(PDF)

S4 Table Top 50 H12 peaks in the DGRP data. Listed are the coordinates of the center of the analysis window with the highest H12 value in a peak, the edge coordinates of each peak, the corresponding H12 and H2/H1 values in the analysis window, the *θ*_A_ inferred for each peak and the associated 95% credible intervals for *θ*_A_ under the constant *N*_*e*_ = 10^6^ and admixture models, Bayes factors calculating the ratio of the likelihood of the data under a soft versus hard sweep model, and the names of the genes overlapping each peak.
(XLS)

S5 Table Test for correlations between locations of the top 50 peaks and inversions in the DGRP data. We performed a two-sided binomial test comparing the observed number of peaks overlapping a given inversion and the distribution of expected number of peaks overlapping an inversion. Inversions were identified by Spencer Koury (personal communication). We tested for correlations with only those inversions that were present in at least two strains. We calculated the expected number of overlapping peaks by assuming a uniform distribution of peaks throughout the genome and calculated the proportion of the genome that each inversion overlapped (‘Probability of overlapping this inversion’). In all but one cases, there was no significant deviation between the observed and expected number of peaks overlapping inversions. Only for In(3R)K did we find a greater than expected number of peaks overlapping the inversion.
(PDF)

S6 Table Test for correlation between haplotypes in cluster groups and haplotypes with inversions. We performed a Chi-square test to determine whether haplotypes comprising cluster groups have greater than expected number of linked inversions on the same chromosome. In this table, we report the *P*-values associated with this test and find that there are no significant enrichments within haplotype groups for inversions that may be linked on the same chromosome.
(PDF)
