# Supplementary material for: Recent Selective Sweeps in North American Drosophila melanogaster Show Signatures of Soft Sweeps
Source: PLoS Genet. 2015 Feb 23;11(2):e1005004. doi: 10.1371/journal.pgen.1005004 (PMC4338236; doi:10.1371/journal.pgen.1005004)
Supplement: S1 Text — (PDF) [file pgen.1005004.s001.pdf]

SUPPLEMENTARY TEXT

Calculation of the 1-per-genome FDR critical value of $H12_o$.

We calculated the critical values, $H12_o$, for the six neutral demographic models and for three different recombination rates, $\rho = 10^{-7}$, 5×10^{-7}, and 10^{-6} cM/bp, based on a 1-per-genome false discovery rate (FDR) criterion. Our test rejects neutrality in favor of a selective sweep when $H12 > H12_o$. The critical values $H12_o$ for rejecting neutrality with a given recombination rate, ρ_0, are conservative for genomic regions with recombination rates $\rho > \rho_0$ (Table 1). Note that $H12_o$ values obtained under models with the lowest recombination rate ($\rho = 10^{-7}$ cM/bp) are substantially higher than $H12_o$ values calculated under models with recombination rates even modestly higher than 10^{-7} cM/bp. Therefore, $H12_o$ values calculated under low recombination rates may be too conservative for most genomic regions. Hence, we used the $H12_o$ value obtained from regions with a low, but not extremely low, $\rho = 5\times10^{-7}$ cM/bp, filtering out all regions with a recombination rate lower than 5×10^{-7} cM/bp from the data.

Robustness of the H12 scan

To ensure that the H12 peaks identified in our genomic scan are robust to any peculiarities of the DGRP data set such as inversions, unaccounted substructure within the data, or sequencing quality, we performed a number of tests. The individual strains of the DGRP data set contain a number of inversions, seven of which are shared across multiple strains (S5A Table) (The locations of inversion breakpoints were identified by Spencer Koury, personal communication). One possibility is that elevated peaks of homozygosity could result from inversions suppressing recombination. To test for this possibility, we performed a binomial two-sided test for enrichment of the top 50 peaks in regions with inversions versus a model of a uniform distribution of the peaks genome-wide. We found no significant enrichment in any inversions except for an inversion on chromosome 3R, In(3R)K (*P*-value=6.44E-06) (S5A Table). We further performed a Chi-square test for a correlation between members of haplotype groups in each peak and haplotypes potentially linked to an inversion on the same chromosome, as inversions have been shown to affect polymorphisms chromosome-wide [1]. We did not find any enrichment for strains bearing inversions in any single haplotype cluster group for the top 50 peaks (S5B Table), suggesting that the enrichment of peaks in the In(3R)K inversion cannot be

attributable to the inversion *per se*. Finally, even after removing regions of the genome overlapping major cosmopolitan inversions, there continues to be an elevation and long tail of H12 values in DGRP data relative to expectations under any neutral demographic model (S6 Fig.).

During our analysis of the DGRP data set, two new data sets based on the same North Carolina population of flies became available: the Drosophila Population Genomics Project (DPGP) data set, which consists of 40 of the original 162 inbred lines in the DGRP data set, and version 2 of the DGRP version data set, comprised of 205 lines including the original 162 lines.

Given the shallower sample depth, we scanned the DPGP data set with a window size of 100 SNPs and found that 16 peaks of the top 50 in the DPGP scan overlap 13 of the top 50 unique peaks in the DPGP scan (S7A Fig.). Ten of these overlapping peaks are among the top 15 peaks in the DGRP scan. We define an overlap of two peaks as an intersection of the edge coordinates of the first and last windows in the two peaks.

We repeated the analysis in the DGRP version 2 data set as well. In the DGRP data set, there are at least five pairs of strains with genome-wide identity by descent (IBD) values > 50% suggesting twin or sibling relationships [2], and three of these complete pairs were among our data set of 145 strains. Since related strains can increase haplotype homozygosity, in our new DGRP v2 scan, we removed one of the members of each closely related pair to ensure that the top 50 H12 peaks are robust to any homozygosity contributed by related pairs of flies. In addition, we removed strains with the most missing data, and down sampled to 145 lines to match the number of strains in the original scan. Forty of the top 50 DGRPv2 peaks overlapped 34 unique peaks among the top 50 peaks in the DGRP scan (S7B Fig.). Since related pairs in a sample of 145 individuals can increase homozygosity at most by $(2/145)^2 = 0.00019$, we did not exclude these lines from the final analysis of the DGRP data.

We scanned the remaining 63 strains that were non-overlapping with the original 145 strains to determine if we could recover the peaks in a completely independent data set, and observed that 12 peaks among the top 50 peaks in this scan overlap 11 unique peaks among the top 50 peaks identified in the DGRP data set (S7C Fig.).

Finally, we sub-sampled the DGRP data set to 40 strains 10 times and plotted the resulting distributions of H12 values (S8 Fig.). In contrast to H12 distributions observed in the six tested neutral demographic models also sampled at 40 strains, there is an elevation and long

tail of genome-wide H12 values, indicating that the elevation in haplotype homozygosity observed in the DGRP data are population-wide and not specific to any subset of the strains.

Estimation of θ_A for the top 50 peaks

The monotonic relationship between the softness of a sweep and both H12 and H2/H1 over the interval ($0.01 < \theta_A < 100$) in Fig. 5 and 10 suggests that these two statistics are informative for the purpose of inferring the softness of a sweep. Here, we estimate the softness of a sweep by varying the parameter θ_A. We developed a Bayesian approach for inferring θ_A by sampling the posterior distribution of θ_A conditional on the observed values $H12_{obs}$ and $H2_{obs}$ /$H1_{obs}$ from a candidate sweep. Given that sampling this true posterior distribution is computationally intractable, we used approximate Bayesian computation (ABC) for our inference procedure. Specifically, we drew θ_A values from a prior distribution, simulated a large data set under each θ_A value, and then kept 1000 parameter values which produce sweeps with H12 and H2/H1 values close to the observed values $H12_{obs}$ and $H2_{obs}$ /$H1_{obs}$ from the candidate sweep (differences <10% for each statistic). From these posterior distributions, we inferred the maximum a posteriori (θ_A^{MAP}) value of the given candidate sweep to estimate its softness (Methods).

We estimated the softness of the top 50 peaks detected in our H12 scan in Fig. 8A by inferring the θ_A^{MAP} value that generates haplotype structure best resembling the spectra observed for each peak using the above ABC procedure. We first considered the $N_e = 10^6$ demographic model and uniform prior distributions for all other parameters: The adaptive mutation rate θ_A took values on [0,100], the selection coefficient (s) on [0,1], the ending partial frequency of the adaptive allele after selection ceased (PF) on [0,1], the time at which selection ended (T_E) on $[0,0.001]\times 4N_e$, and the recombination rate (ϱ) on an interval containing the observed recombination rate at each peak (see Methods).

The posterior distributions of θ_A and the estimates of θ_A^{MAP} for the top nine peaks obtained by our procedure are shown in S10A Fig. The distribution of θ_A^{MAP} values for all top 50 peaks is shown in S10B Fig. S4 Table lists all θ_A^{MAP} values and their 95% confidence intervals. The minimum θ_A^{MAP} value among all 50 top peaks is $\theta_A^{MAP} = 6.8$, which is obtained for the peak centered at *Cyp6g1*.

We also estimated θ_A^{MAP} for our top 50 peaks under the admixture model proposed by Duchen *et al*. [3] to determine the effect of admixture on our estimates (Methods). S10A Fig. shows the comparison of the posterior distributions of θ_A inferred under the constant $N_e = 10^6$ and admixture models for the top nine peaks. The posterior distributions of θ_A under the admixture model tends to have a smaller variance than under the constant $N_e = 10^6$ model. S10B Fig. and S4 Table show that θ_A^{MAP} estimates of the top nine peaks for the two models are similar, but slightly higher under the admixture model as compared to the constant $N_e = 10^6$ model. This suggests that the θ_A^{MAP} estimates under the constant $N_e = 10^6$ model are in fact conservative in estimating the softness of each peak.

SUPPLEMENTARY REFERENCES

1. Corbett-Detig RB, Hartl DL (2012) Population genomics of inversion polymorphisms in *Drosophila melanogaster*. PLoS Genetics 8: e1003056.
2. Cridland JM, Macdonald SJ, Long AD, Thornton KR (2013) Abundance and distribution of transposable elements in two Drosophila QTL mapping resources. Molecular Biology and Evolution 30: 2311-2327.
3. Duchen P, Zivkovic D, Hutter S, Stephan W, Laurent S (2013) Demographic inference reveals African and European admixture in the North American *Drosophila melanogaster* population. Genetics 193: 291-301.
