# Supplementary figures and images for: Recent Selective Sweeps in North American Drosophila melanogaster Show Signatures of Soft Sweeps
Source: PLoS Genet. 2015 Feb 23;11(2):e1005004. doi: 10.1371/journal.pgen.1005004 (PMC4338236; doi:10.1371/journal.pgen.1005004)

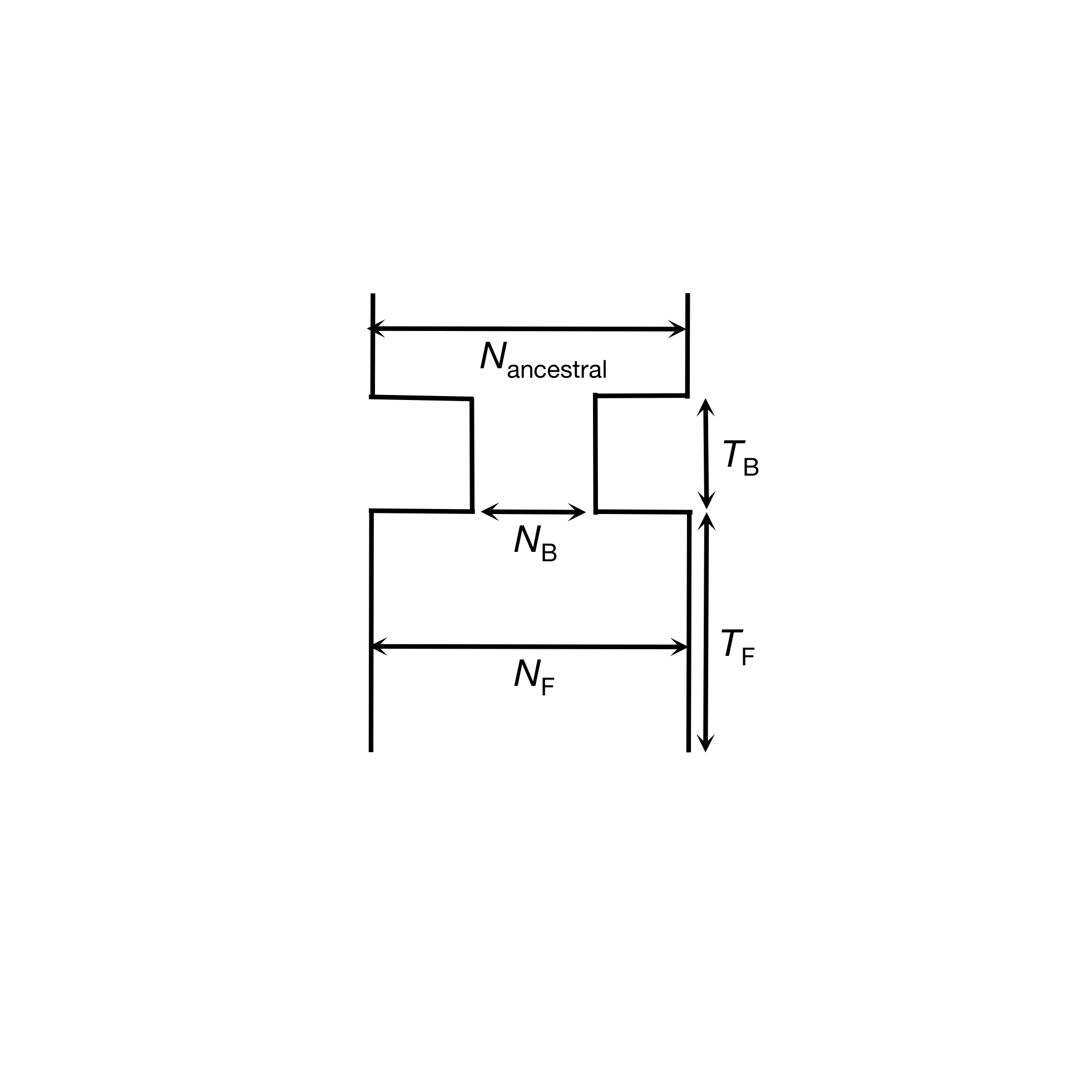

Supplement: S1 Fig — The inferred parameters were the size of the final population (NF), the duration of the bottleneck (TB), and the time after the bottleneck (TF). Investigated bottleneck sizes ranged from NB = 0.002 to NB = 0.4 (see S2 Table). NB = 0.002 represents the population size of the bottleneck inferred for European flies by Li and Stephan (2006) [64], whereas NB = 0.4 represents a comparatively shallow population size reduction. (TIF) [file pgen.1005004.s002.tif]

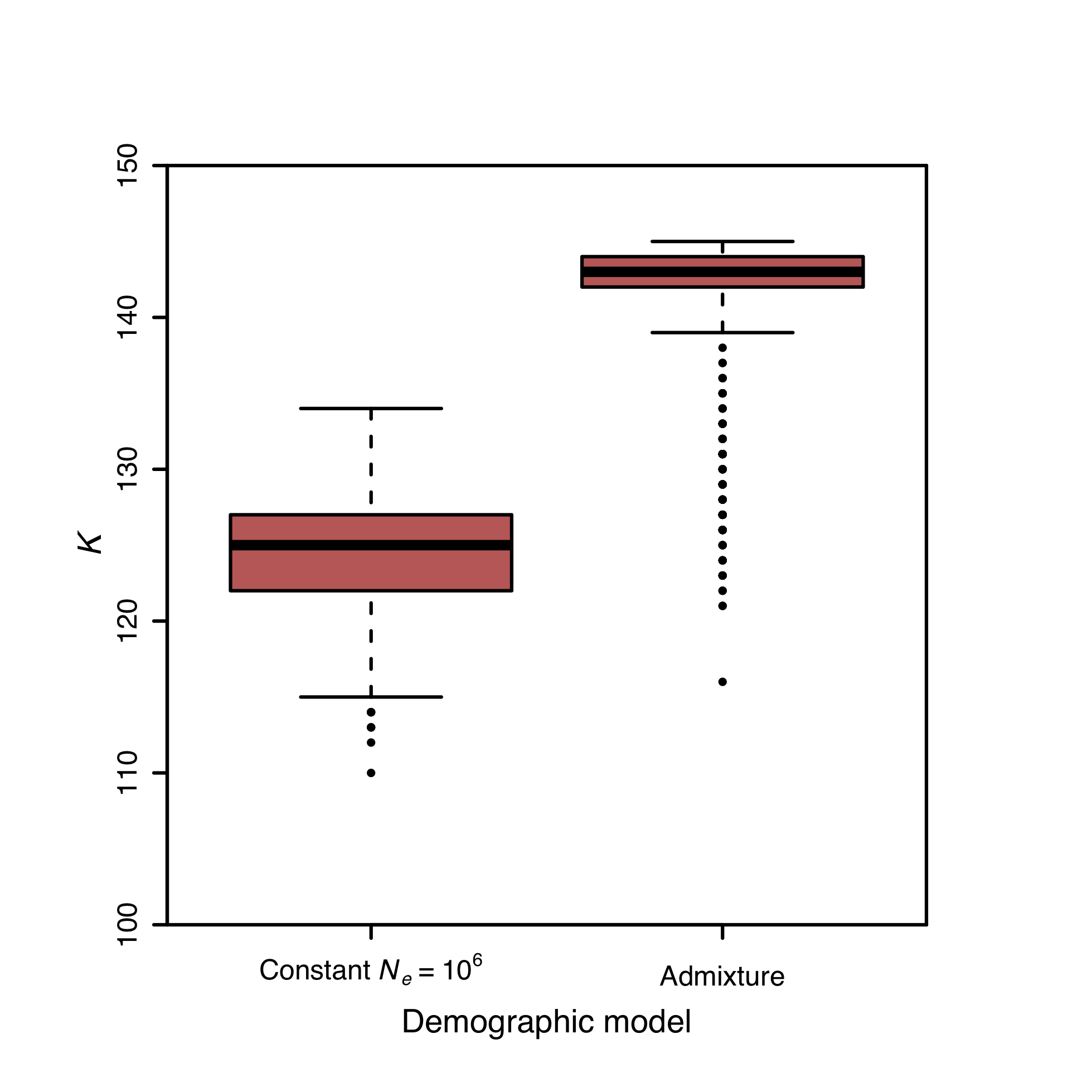

Supplement: S2 Fig — We observe a significantly higher number of unique haplotypes (K) in neutral simulations of admixture as compared to a constant N e scenario. Here we plot distributions of K in a sample of haplotypes drawn from the North American deme in the admixture model in Fig. 1 and a constant N e = 106 model. In each scenario, 1000 simulations were performed. (TIF) [file pgen.1005004.s003.tif]

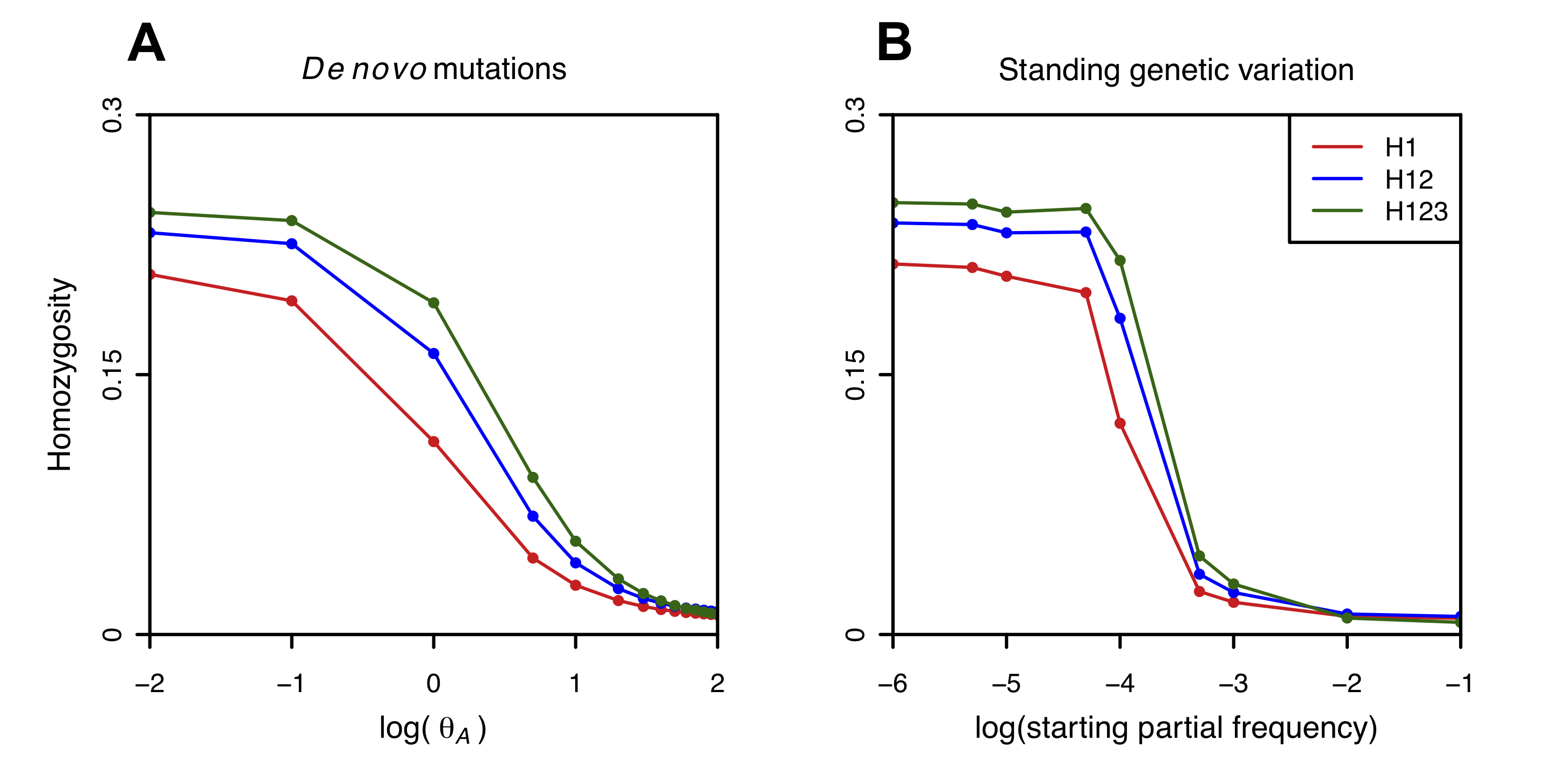

Supplement: S3 Fig — Homozygosity values were measured in simulated sweeps arising from (A) de novo mutations with θ A values ranging from 10–2 to 102 and (B) SGV with starting frequencies ranging from 10–6 to 10–1. Sweeps were simulated under a constant N e = 106 demographic model with a recombination rate of 5×10^–7^ cM/bp, selection coefficient of s = 0.01, and ending partial frequency of the adaptive allele after selection ceased, PF = 0.5. Each data point was averaged over 1000 simulations. H1, H12, and H123 values all decline rapidly as the softness of a sweep increases. H12 modestly augments our ability to detect a sweep as long as the sweep is not too soft or too old. H123 has marginally better ability to detect selective sweeps as compared to H12. (TIF) [file pgen.1005004.s004.tif]

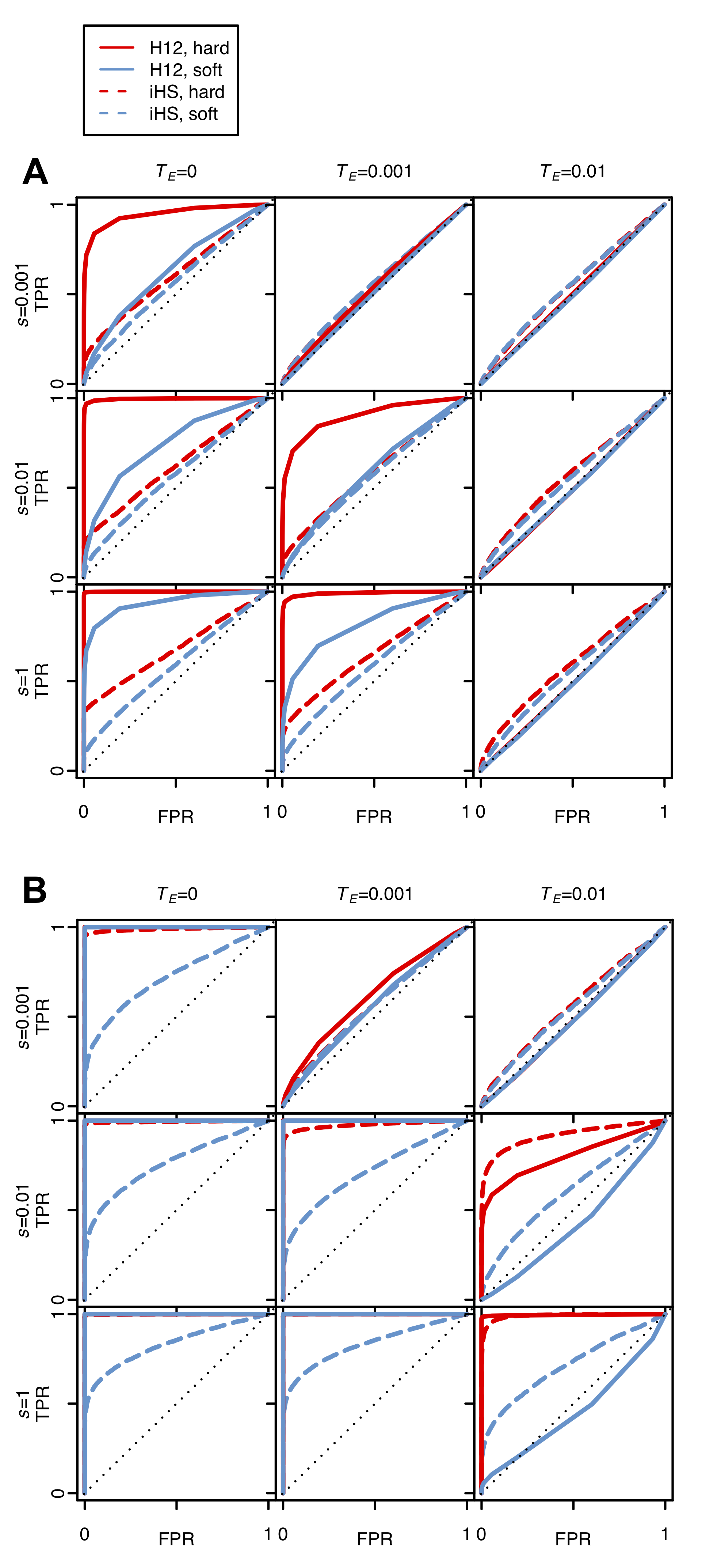

Supplement: S4 Fig — Same as Fig. 6, except ending partial frequencies of the adaptive allele after selection ceased are PF = 0.1 in (A) and PF = 0.9 in (B). (TIF) [file pgen.1005004.s005.tif]

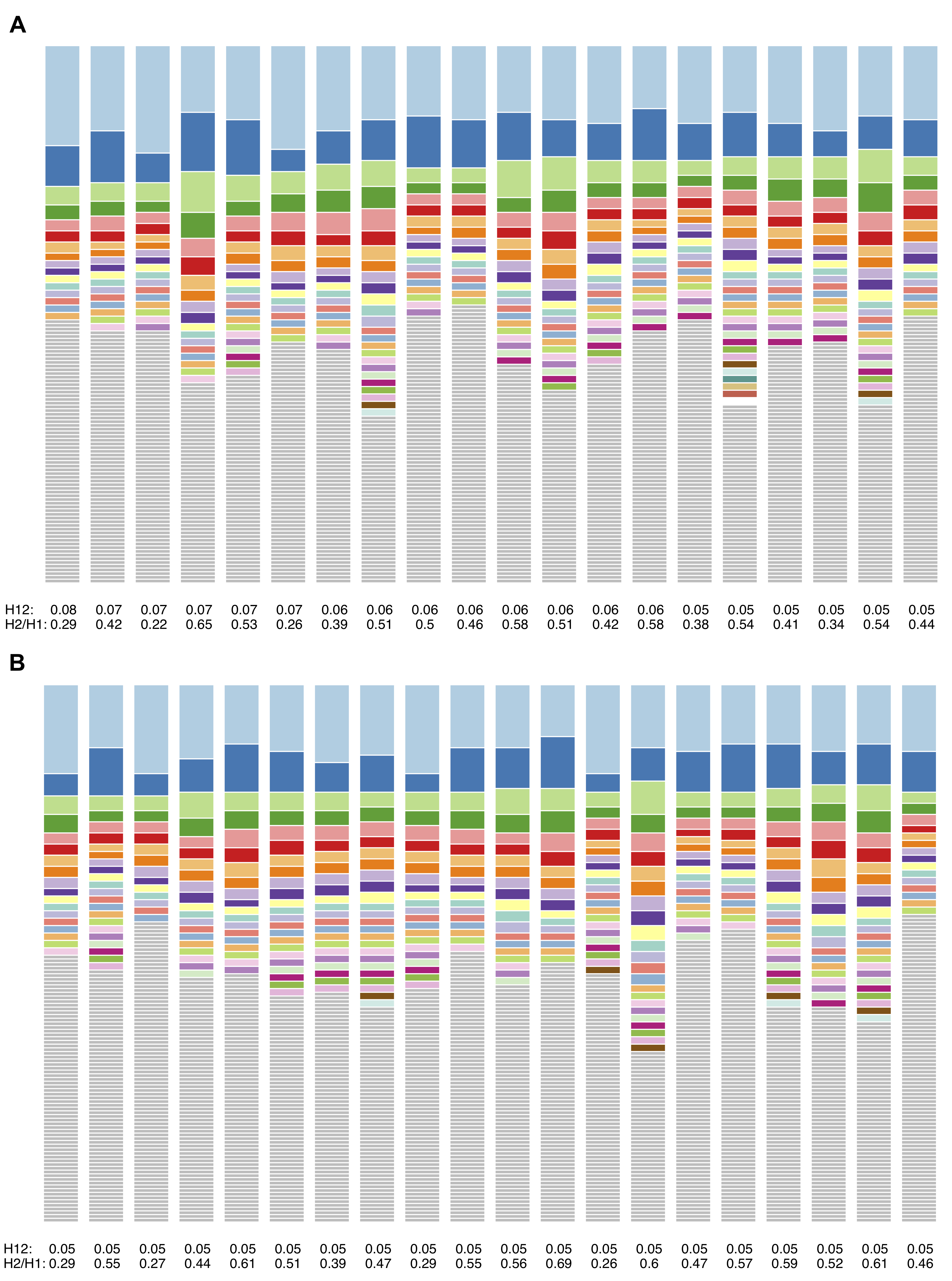

Supplement: S5 Fig — Same as Fig. 9, except plotted are haplotype frequency spectra for the (A)11th-30th and the (B) 31st—50th peaks in the DGRP scan. (TIF) [file pgen.1005004.s006.tif]

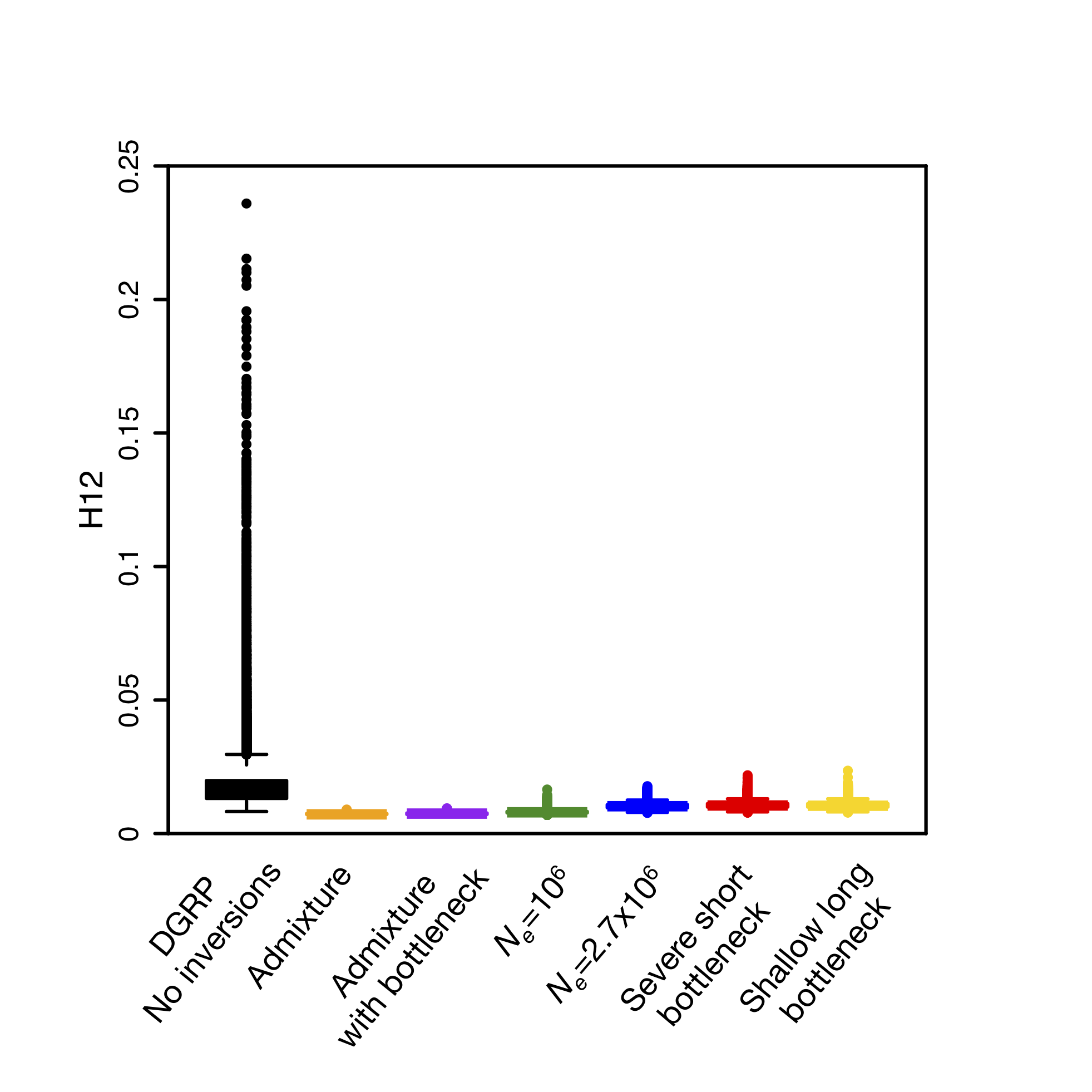

Supplement: S6 Fig — Similar to Fig. 7, except here regions overlapping major cosmopolitan inversions are excluded from the distribution of H12 values in DGRP data. There is a long tail and elevation of H12 values in DGRP data as compared to expectations under any neutral demographic model tested. (TIF) [file pgen.1005004.s007.tif]

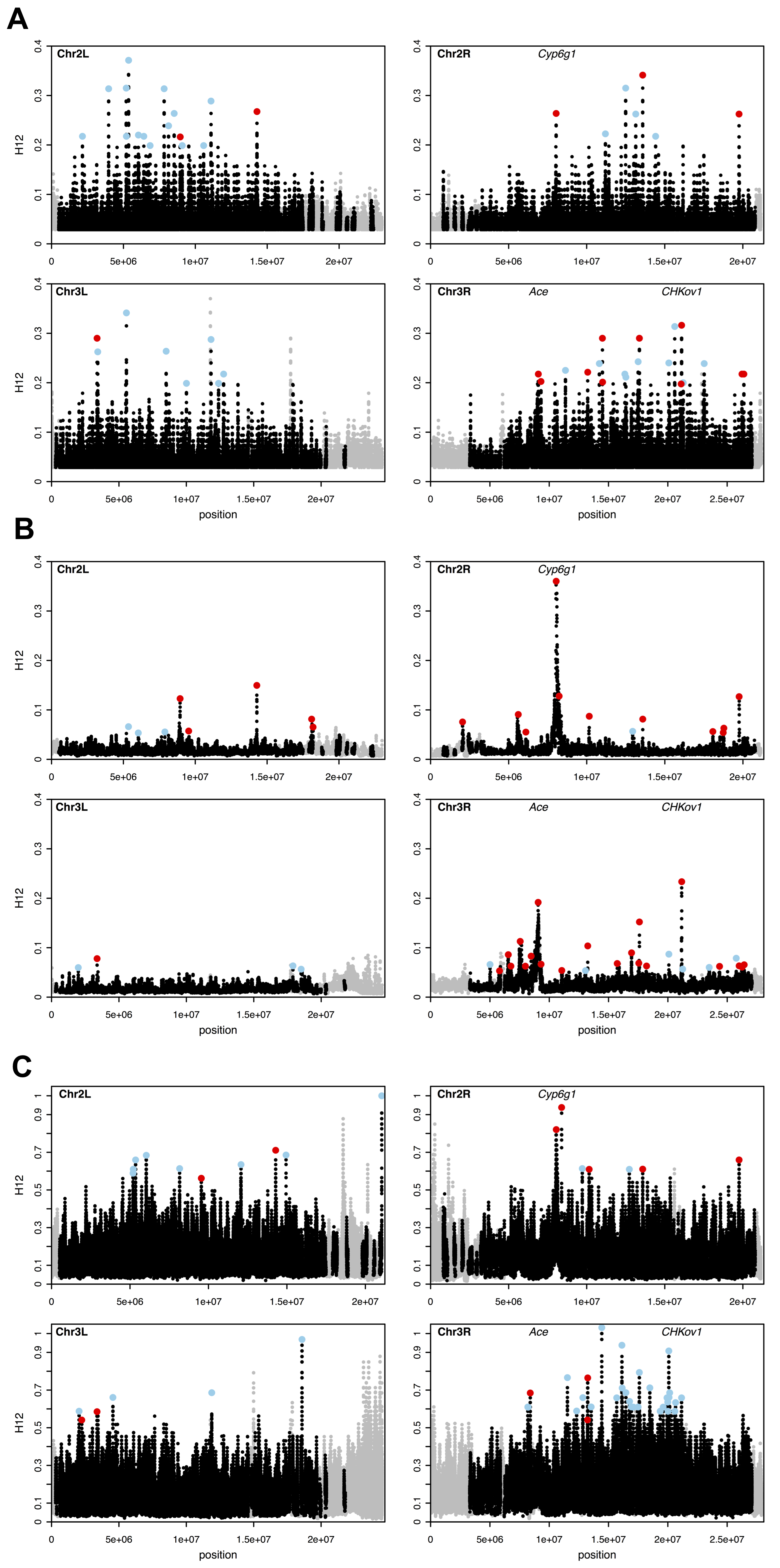

Supplement: S7 Fig — We reran the H12 scan in three data sets: (A) DPGP data, (B) DGRP version 2 data set, and (C) the 63 DGRP version 2 strains that do not overlap the 145 strains used in the original DGRP scan. Blue and red points highlight the top 50 most extreme peaks with high H12 values relative to the median H12 value in the scan. Red points indicate peaks among the top 50 in each scan that overlap the top 50 peaks observed in the original DGRP scan. In (A), 16 peaks overlap, in (B), 40 peaks overlap, and in (C), 12 peaks overlap. Most of the overlapping peaks are among the top ranking peaks in the DGRP scan. We identify the three well-characterized cases of selection in D. melanogaster at Ace, CHKov1, and Cyp6g1 in all three scans. (TIF) [file pgen.1005004.s008.tif]

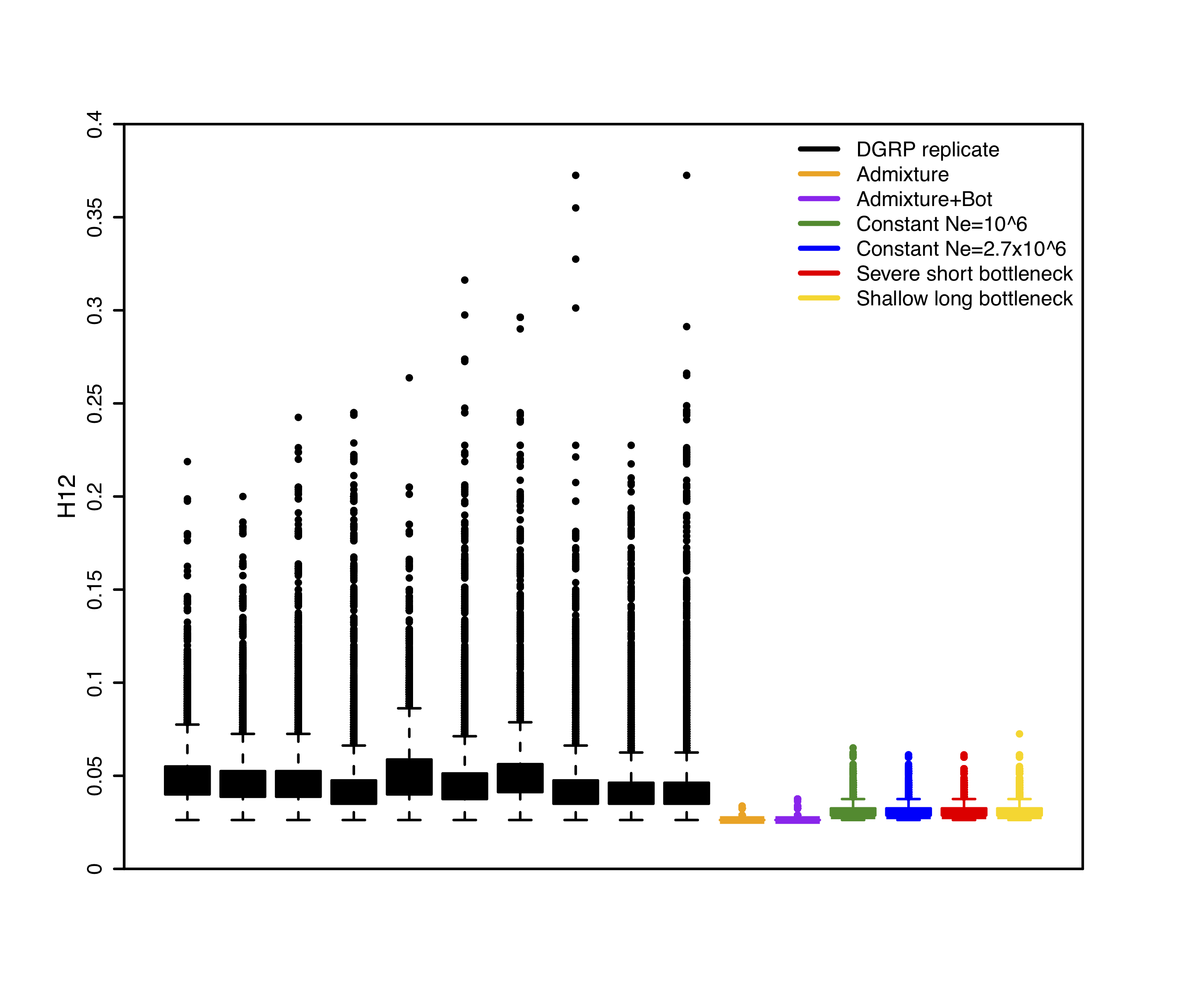

Supplement: S8 Fig — DGRP strains were downsampled to 40 strains 10 times and the resulting distributions of H12 were plotted (black). In contrast to expectations under any neutral demographic model tested with a sample size of 40, all samples of 40 strains have elevated H12 values and a long tail. This indicates that the elevation of homozygosity values observed in DGRP data in Fig. 7 is driven by a population-wide signal and not by any sub-population. (TIF) [file pgen.1005004.s009.tif]

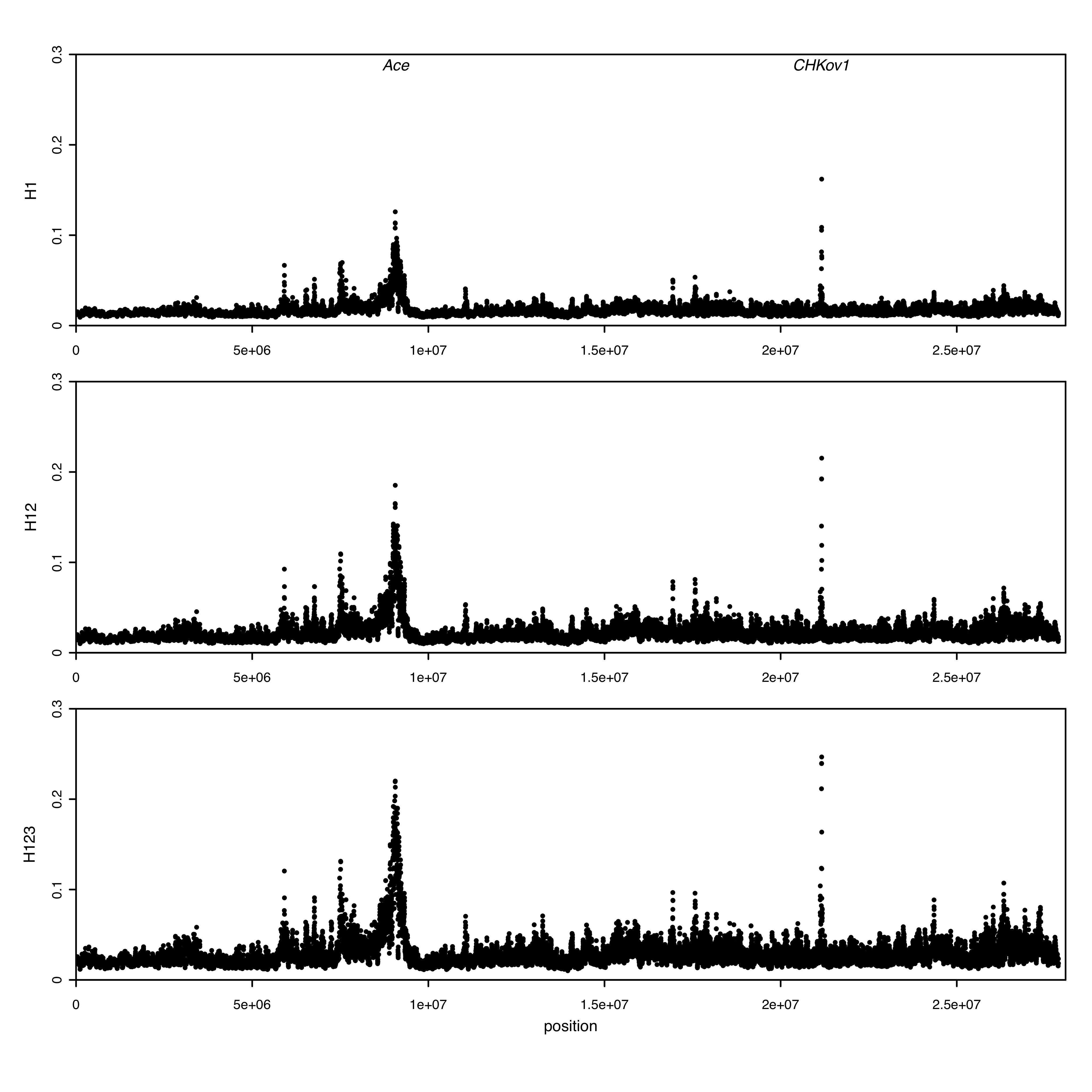

Supplement: S9 Fig — All statistics are able to identify similar peaks. The known cases of adaptation at Ace and CHKov1 have more pronounced peaks under H12 and H123. (TIF) [file pgen.1005004.s010.tif]

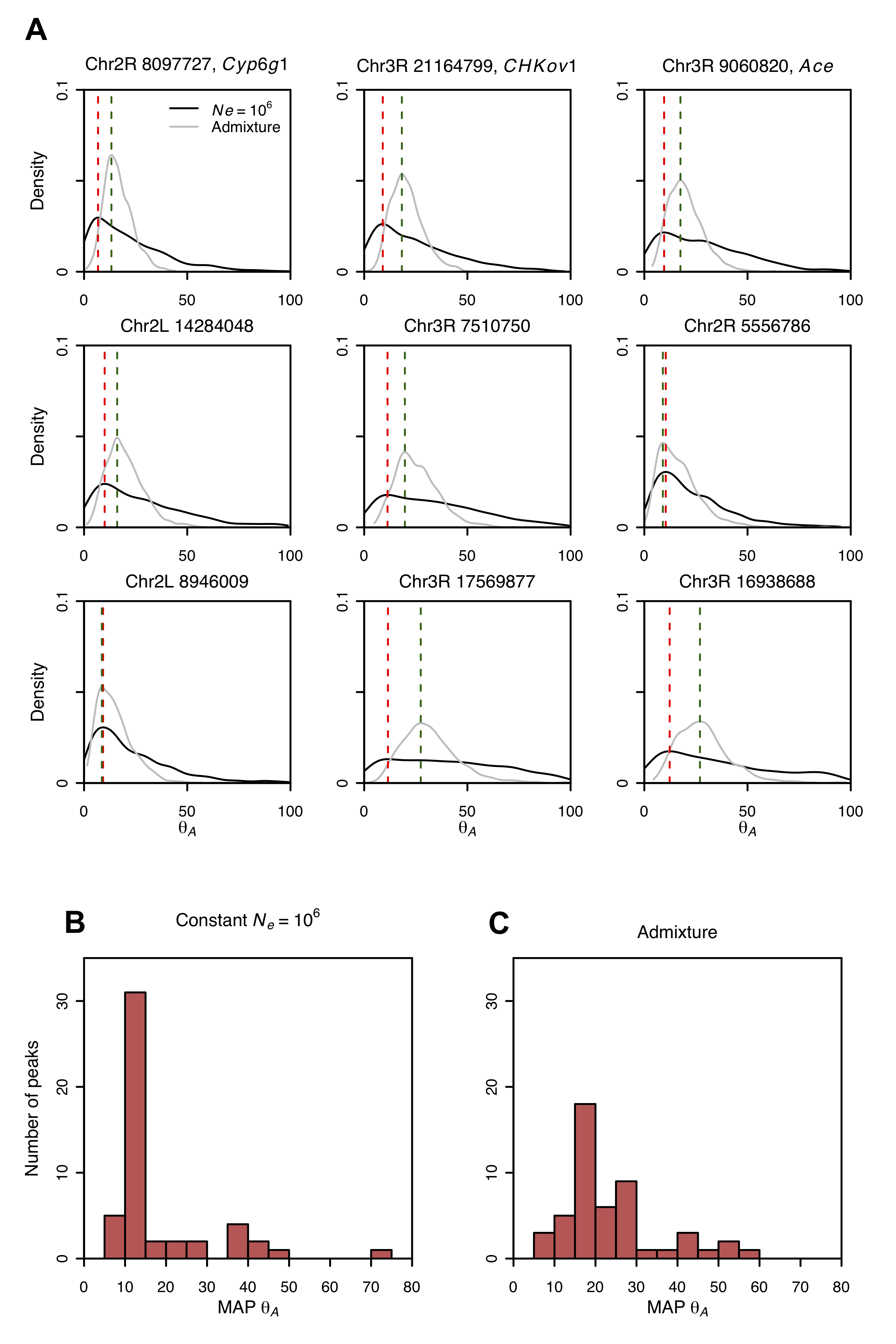

Supplement: S10 Fig — (A) Posterior distributions of θ A measured under the constant N e = 106 model and the admixture model (black and grey lines, respectively) and the corresponding θ A MAP estimates (dashed red and green lines, respectively) for the top nine peaks. (B) Distribution of θ A MAP values inferred under the constant N e = 106 model for the top 50 peaks. (C) Corresponding distribution under the admixture model. The distribution of θ A MAP peaks around θ A = 10 under the constant N e = 106 model and peaks at a slightly higher value under the admixture model, suggesting that the constant N e = 106 model may be conservative for the purposes of inferring the softness of a sweep. (TIF) [file pgen.1005004.s011.tif]

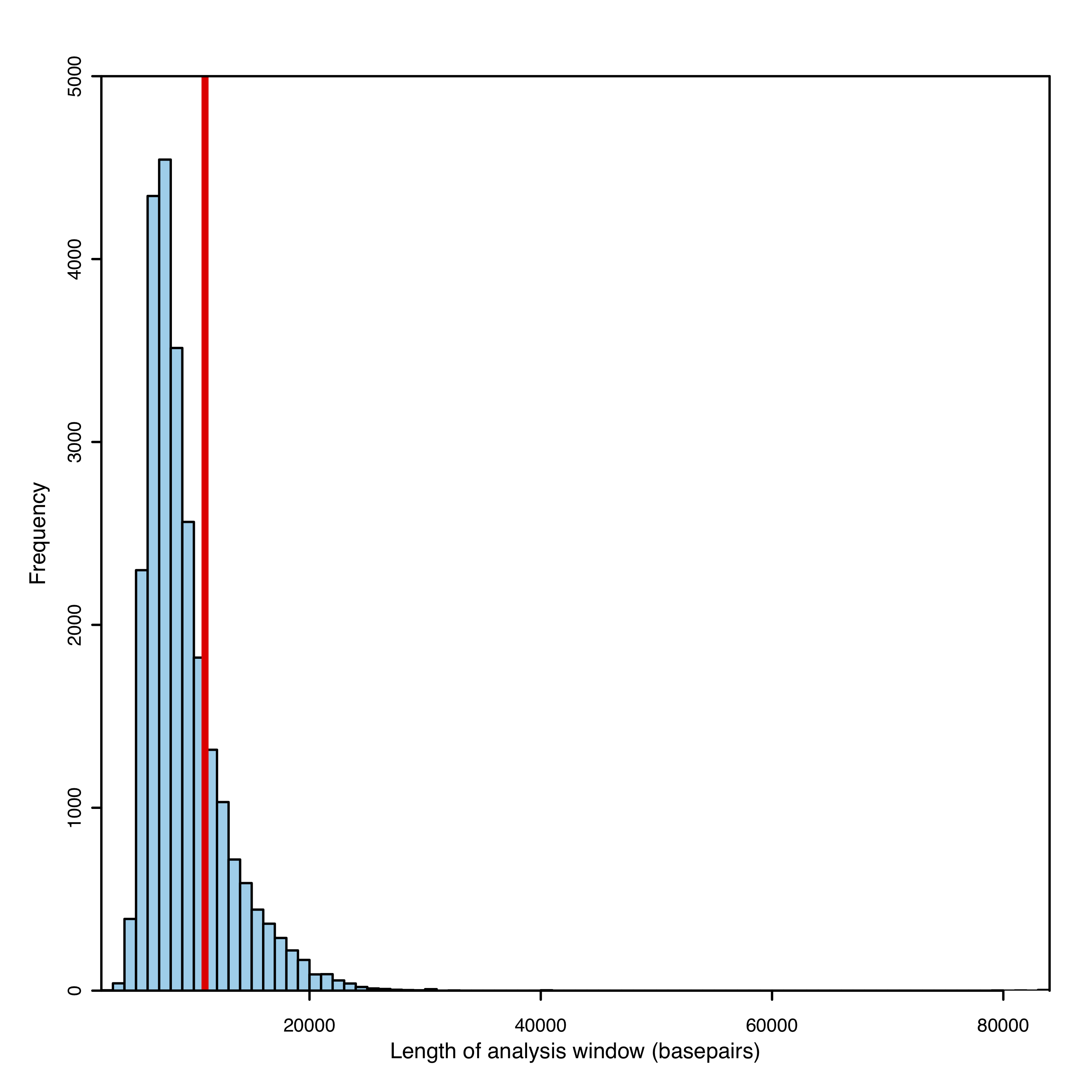

Supplement: S11 Fig — To confirm that the the analysis windows with the highest H12 values for our top 50 peaks are not unusually short, we plotted the distribution of window lengths for randomly chosen analysis windows genome-wide. For each of the top 50 peaks, 500 analysis windows with recombination rates within 10% of the observed recombination rate in analysis window with the highest H12 values were drawn randomly. A total of 25,000 windows comprise the distribution below. Plotted in red is the mean window length of the analysis windows for the top 50 peaks. The left tail empirical P-value is 0.77. (TIF) [file pgen.1005004.s012.tif]
