# Supplementary material for: Recent Selective Sweeps in North American Drosophila melanogaster Show Signatures of Soft Sweeps
Source: PLoS Genet. 2015 Feb 23;11(2):e1005004. doi: 10.1371/journal.pgen.1005004 (PMC4338236; doi:10.1371/journal.pgen.1005004)
Supplement: S1 Table — Point estimates were calculated by Pablo Duchen (personal communication). All population sizes are in units of N Ac. In the admixture model (A), N Ac = 4,975,360, and in the admixture with bottleneck model (B), N Aa = 3,100,520. All times are in units 4N Ac. (PDF) [file pgen.1005004.s013.pdf]

S1 Table. Parameter values used for simulations of admixture models from Fig. 1. Point estimates were calculated by Pablo Duchen (personal communication). All population sizes are in units of $N_{Ac.}$ In the admixture model (A), N_{Ac}=4,975,360, and in the admixture with bottleneck model (B), N_{Aa}=3,100,520. All times are in units $4N_{Ac}$.

A) Admixture model

Parameter	Symbol	Value
Ancient size Africa	N_{Aa}	1.049994
Time of bottleneck Africa	T_A	0.1192512
Severity of bottleneck Africa	sev_A	0.21
Current size Africa	N_{Ac}	1
Time of admixture	T_{adm}	7.263e-05
Proportion of European admixture	$prop_{adm}$	0.85
Time of split Africa-Europe	T_{AE}	0.009798
Ancient size North America	N_{Na}	0.0005048653
Current size North America	N_{Nc}	3.2127
Ancient size Europe	N_{Ea}	0.003413308
Current size Europe	N_{Ec}	0.6276

B) Admixture with bottleneck model

Parameter	Symbol	Value
Ancient size Africa	N_{Aa}	1.0401
Time of bottleneck Africa	T_A	0.03241136
Severity of bottleneck Africa	sev_A	0.615123
Current size Africa	N_{Ac}	1
Time of admixture	T_{adm}	3.757037e-05
Proportion of European admixture	$prop_{adm}$	0.871794
Time of split Africa-Europe	T_{AE}	0.006037894
Current size North America	N_{Nc}	2.968357
Ancient size Europe	N_{Ea}	0.004306807
Current size Europe	N_{Ec}	0.7318321
