# Supplementary material for: Recent Selective Sweeps in North American Drosophila melanogaster Show Signatures of Soft Sweeps
Source: PLoS Genet. 2015 Feb 23;11(2):e1005004. doi: 10.1371/journal.pgen.1005004 (PMC4338236; doi:10.1371/journal.pgen.1005004)
Supplement: S2 Table — Shown are parameter estimates for six simple bottleneck scenarios fit to short intron data in DGRP inferred by DaDi [47] and the corresponding log likelihoods for each model (LL). For all inferred models, the bottleneck sizes (N B) were fixed at values as specified in the table. All population size estimates are in terms of units 4*Ne ancestral, and all time estimates are in terms of units 2*Ne ancestral. Values of θ exp were measured for each inferred demographic models and are a function of the number of base pairs (738,024) used to generate the SFS. Note that N B = 0.002 represents the population size of the bottleneck inferred by Li and Stephan (2006) [64] and N B = 0.029 is the population size of the bottleneck inferred by Thornton and Andolfatto (2006) [46]. We ultimately chose to use the short severe bottleneck model (N B = 0.002, T B = 0.0002) and shallow long bottleneck model (N B = 0.4, T B = 0.0560) because all models fit the data equally well and these two models represent the extreme ends of the range of models tested. See S3 Table for a comparison of the fit of the severe short and shallow long bottleneck models to short intron data in terms of S and π. (PDF) [file pgen.1005004.s014.pdf]

S2 Table. Demographic parameters inferred by DaDi for simple bottleneck scenarios. Shown are parameter estimates for six simple bottleneck scenarios fit to short intron data in DGRP inferred by DaDi [47] and the corresponding log likelihoods for each model (LL). For all inferred models, the bottleneck sizes (N_B) were fixed at values as specified in the table. All population size estimates are in terms of units $4^*Ne_{\text{ancestral}}$, and all time estimates are in terms of units $2^*Ne_{\text{ancestral}}$. Values of θ_{exp} were measured for each inferred demographic models and are a function of the number of base pairs (738,024) used to generate the SFS. Note that N_B=0.002 represents the population size of the bottleneck inferred by Li and Stephan (2006) [64] and N_B=0.029 is the population size of the bottleneck inferred by Thornton and Andolfatto (2006) [46]. We ultimately chose to use the short severe bottleneck model (N_B=0.002, T_B=0.0002) and shallow long bottleneck model (N_B=0.4, T_B=0.0560) because all models fit the data equally well and these two models represent the extreme ends of the range of models tested. See S3 Table for a comparison of the fit of the severe short and shallow long bottleneck models to short intron data in terms of S and π.

N_B	N_F	T_B	T_F	LL	θ_{exp}
0.002	0.601	0.0002	0.33	-285.77	10023.95
0.029	0.683	0.0021	0.18	-285.67	11337.58
0.05	0.682	0.0037	0.18	-285.67	10024.82
0.1	0.682	0.0080	0.18	-285.67	10027.46
0.2	0.682	0.0186	0.18	-285.67	10034.67
0.4	0.679	0.0560	0.16	-285.68	10069.48
