# Supplementary material for: Recent Selective Sweeps in North American Drosophila melanogaster Show Signatures of Soft Sweeps
Source: PLoS Genet. 2015 Feb 23;11(2):e1005004. doi: 10.1371/journal.pgen.1005004 (PMC4338236; doi:10.1371/journal.pgen.1005004)
Supplement: S3 Table — Estimates of S and π were averaged over 30,000 simulations of 10,000 bps for each demographic model. S and π estimates in DGRP short intron data were measured to be 5.8% and 1.2% per bp, respectively. (PDF) [file pgen.1005004.s015.pdf]

S3 Table. *S* and *π* measured in neutral demographic models of North American *Drosophila*. Estimates of *S* and *π* were averaged over 30,000 simulations of 10,000 bps for each demographic model. *S* and *π* estimates in DGRP short intron data were measured to be 5.8% and 1.2% per bp, respectively.

**Demographic model**	***S*/bp**	***π*/bp**
Admixture	5.8%	1.1%
Admixture + bottleneck	5.6%	1.3%
Constant Ne=10^6	2.3%	0.4%
Constant Ne=2.7x10^6	5.8%	1.1%
Severe short bottleneck	5.7%	1.1%
Shallow long bottleneck	5.5%	1.1%
