# Supplementary material for: Recent Selective Sweeps in North American Drosophila melanogaster Show Signatures of Soft Sweeps
Source: PLoS Genet. 2015 Feb 23;11(2):e1005004. doi: 10.1371/journal.pgen.1005004 (PMC4338236; doi:10.1371/journal.pgen.1005004)
Supplement: S5 Table — We performed a two-sided binomial test comparing the observed number of peaks overlapping a given inversion and the distribution of expected number of peaks overlapping an inversion. Inversions were identified by Spencer Koury (personal communication). We tested for correlations with only those inversions that were present in at least two strains. We calculated the expected number of overlapping peaks by assuming a uniform distribution of peaks throughout the genome and calculated the proportion of the genome that each inversion overlapped (‘Probability of overlapping this inversion’). In all but one cases, there was no significant deviation between the observed and expected number of peaks overlapping inversions. Only for In(3R)K did we find a greater than expected number of peaks overlapping the inversion. (PDF) [file pgen.1005004.s017.pdf]

S5 Table. Test for correlations between locations of the top 50 peaks and inversions in the DGRP data. We performed a two-sided binomial test comparing the observed number of peaks overlapping a given inversion and the distribution of expected number of peaks overlapping an inversion. Inversions were identified by Spencer Koury (personal communication). We tested for correlations with only those inversions that were present in at least two strains. We calculated the expected number of overlapping peaks by assuming a uniform distribution of peaks throughout the genome and calculated the proportion of the genome that each inversion overlapped ('Probability of overlapping this inversion'). In all but one cases, there was no significant deviation between the observed and expected number of peaks overlapping inversions. Only for In(3R)K did we find a greater than expected number of peaks overlapping the inversion.

Inversion	Number of overlapping peaks	Probability of overlapping this inversion	*P*-value (*P*-binom two sided)	Interpretation
all inversions	40	0.879	0.123	Insignificant
In(2L)t	4	0.164	0.127	Insignificant
In(2R)ns	1	0.065	0.259	Insignificant
In(2R)nc	3	0.037	0.434	Insignificant
In(3L)P	1	0.188	0.001	Lower than expected
In(3R)Mo	7	0.163	0.848	Insignificant
In(3R)K	16	0.100	6.44E-06	Greater than expected
In(3R)P	11	0.136	0.096	Insignificant
