# Supplementary material for: Recent Selective Sweeps in North American Drosophila melanogaster Show Signatures of Soft Sweeps
Source: PLoS Genet. 2015 Feb 23;11(2):e1005004. doi: 10.1371/journal.pgen.1005004 (PMC4338236; doi:10.1371/journal.pgen.1005004)
Supplement: S6 Table — We performed a Chi-square test to determine whether haplotypes comprising cluster groups have greater than expected number of linked inversions on the same chromosome. In this table, we report the P-values associated with this test and find that there are no significant enrichments within haplotype groups for inversions that may be linked on the same chromosome. (PDF) [file pgen.1005004.s018.pdf]

Chr	Position	ChiSqVal	Df	p-value
Chr2R	8097727	21.87	24	0.587
Chr3R	21164799	19.86	54	1.000
Chr3R	9060820	54.57	54	0.453
Chr2L	14284048	12.92	18	0.796
Chr3R	7510750	42.92	54	0.861
Chr2R	5556786	41.63	32	0.119
Chr2L	8946009	8.20	15	0.916
Chr3R	17569877	34.75	66	0.999
Chr3R	16938688	22.79	72	1.000
Chr2L	18132779	19.82	22	0.594
Chr2R	19764552	19.46	32	0.960
Chr2R	2043155	17.29	36	0.996
Chr3R	6766917	47.40	57	0.814
Chr3R	26334451	42.64	54	0.868
Chr2R	6196252	8.47	46	1.000
Chr2L	18196971	13.44	17	0.706
Chr3R	7892320	34.23	57	0.993
Chr3R	26036261	50.56	75	0.986
Chr3R	18175477	67.60	57	0.159
Chr2R	18097586	12.03	34	1.000
Chr3R	24353929	19.86	63	1.000
Chr2R	5735958	14.76	44	1.000
Chr2L	20088273	14.56	23	0.910
Chr3R	17917391	46.48	63	0.941
Chr2R	18779397	20.68	42	0.998
Chr2R	18723092	32.25	58	0.998
Chr2L	9543046	14.60	21	0.842
Chr3R	11057699	41.41	63	0.984
Chr3R	26932837	50.68	75	0.986
Chr2R	13587388	11.86	34	1.000
Chr3L	3379750	7.26	18	0.988
Chr3R	15339462	65.51	69	0.597
Chr3R	18556910	42.90	45	0.561
Chr2L	14851029	8.85	20	0.985
Chr3R	15864238	15.20	57	1.000
Chr2L	8317289	12.89	23	0.954
Chr3R	8471637	56.62	69	0.857
Chr3R	27035947	57.29	75	0.936
Chr3R	6517364	21.12	69	1.000
Chr3R	17868544	24.18	54	1.000
Chr3R	26272089	45.50	60	0.917
Chr2R	2453765	14.25	34	0.999
Chr2R	6101046	139.46	48	0.000
Chr3R	13245371	73.64	72	0.424
Chr2R	10140367	10.13	40	1.000
Chr2L	4156488	15.46	18	0.630
Chr3R	15434756	30.14	75	1.000
Chr3R	14491226	34.20	63	0.999
Chr3L	2243951	35.78	25	0.075
Chr3R	5814615	16.85	51	1.000
